# An Emerging Mycoplasma Associated with Trichomoniasis, Vaginal Infection and Disease

**DOI:** 10.1371/journal.pone.0110943

**Published:** 2014-10-22

**Authors:** Jennifer M. Fettweis, Myrna G. Serrano, Bernice Huang, J. Paul Brooks, Abigail L. Glascock, Nihar U. Sheth, Vaginal Microbiome Consortium, Jerome F. Strauss, Kimberly K. Jefferson, Gregory A. Buck

**Affiliations:** 1 Department of Microbiology and Immunology, Virginia Commonwealth University, Richmond, Virginia, United States of America; 2 Center for the Study of Biological Complexity, Virginia Commonwealth University, Richmond, Virginia, United States of America; 3 Department of Statistical Sciences and Operations Research, Virginia Commonwealth University, Richmond, Virginia, United States of America; 4 Department of Obstetrics and Gynecology, Virginia Commonwealth University, Richmond, Virginia, United States of America; Miami University, United States of America

## Abstract

Humans are colonized by thousands of bacterial species, but it is difficult to assess the metabolic and pathogenic potential of the majority of these because they have yet to be cultured. Here, we characterize an uncultivated vaginal mycoplasma tightly associated with trichomoniasis that was previously known by its 16S rRNA sequence as “Mnola.” In this study, the mycoplasma was found almost exclusively in women infected with the sexually transmitted pathogen *Trichomonas vaginalis*, but rarely observed in women with no diagnosed disease. The genomes of four strains of this species were reconstructed using metagenome sequencing and assembly of DNA from four discrete mid-vaginal samples, one of which was obtained from a pregnant woman with trichomoniasis who delivered prematurely. These bacteria harbor several putative virulence factors and display unique metabolic strategies. Genes encoding proteins with high similarity to potential virulence factors include two collagenases, a hemolysin, an O-sialoglycoprotein endopeptidase and a *feoB*-type ferrous iron transport system. We propose the name “*Candidatus* Mycoplasma girerdii” for this potential new pathogen.

## Introduction

Application of next-generation sequencing to the study of the human microbiome is rapidly transforming our understanding of the diversity of the microbial communities that inhabit the human body [1]. However, progress towards the identification of specific microbiome signatures or specific organisms with strong links to disease states has proven elusive. We characterize a new vaginal mycoplasma species “*Candidatus* Mycoplasma girerdii”, previously identified only by its 16S rRNA sequence [2], that exhibits a strong and unique association with the sexually transmitted pathogen *Trichomonas vaginalis*.

Organisms of the *Mycoplasma* and *Ureaplasma* genera are collectively referred to as mycoplasmas. They lack cell walls, have small genomes and are often dependent on their hosts. Mycoplasmas of the female urogenital tract are associated with bacterial vaginosis (BV), pelvic inflammatory disease, preterm labor and preterm birth [3], [4]. These mycoplasmas are among the most common organisms to invade the amniotic cavity, and their carriage is associated with chorioamnionitis in preterm premature rupture of membranes (PPROM) [5]. Moreover, uncultivated and uncharacterized bacterial species also invade the amniotic cavity and likely impact pregnancy outcome [6]. Mycoplasmas can induce inflammatory cytokines in the host [4], and they are more prevalent in the vaginal flora of HIV-infected women [7]. Ureaplasmas have been associated with complications during pregnancy [4], *M. genitalium* with pelvic inflammatory disease, cervicitis, endometritis and salpingitis [8], and *M. hominis* with BV [3] and trichomoniasis [9], [10]. *M. genitalium* is an emerging sexually-transmitted infection, which causes nongonococcal urethritis in men. Despite these associations with disease, *M. hominis* and *Ureaplasma* are also common in apparently healthy women. *T. vaginalis* causes trichomoniasis, the most common non-viral sexually transmitted infection worldwide [11]. Trichomoniasis often accompanies low levels of lactobacilli [12] and BV, and has been implicated in an array of pregnancy complications [13], [14]. Although the extracellular eukaryotic parasite binds to vaginal epithelial cells and is hemolytic [15], the mechanisms of its pathogenesis remain enigmatic.

Martin *et al*. [2] recently described a 16S rRNA sequence from an unknown *Mycoplasma*, which they called “Mnola”, in vaginal secretions and found it to be strongly associated with presence of *T. vaginalis* in the study. Shortly thereafter, Costello *et al.* [16] reported a phylotype with a 16S rRNA sequence exhibiting ∼99% identity to that of the new mycoplasma described herein and by Martin *et al.* [2] as a predominant taxon in the oral sample of a low birth-weight infant (24.5 wks) and speculated it may have been acquired by vertical transmission during delivery. Hyman *et al*. also subsequently identified a partial 16S rRNA sequence from a vaginal sample of a woman who delivered full term that is 99% similar to “*Ca*. M. girerdii” [17]. We independently identified this phylotype and its association with *T. vaginalis*, first reported by Martin *et al*. In the current work, we confirm and extend the characterization of this new *Mycoplasma* using metagenomic strategies, present the genomic sequences of four independently identified strains, one of which was isolated from a pregnant woman who subsequently delivered preterm, and we propose “*Candidatus* Mycoplasma girerdii” for its name.

## Results and Discussion

As part of the Vaginal Human Microbiome Project at Virginia Commonwealth University, we generated 16S rRNA gene-based microbiome profiles for 1,361 mid-vaginal samples collected from women visiting outpatient clinics and an additional 110 samples collected in a labor and delivery unit [18]. Our analyses revealed a novel mycoplasma phylotype that represented the most abundant bacterium observed in 25 mid-vaginal samples (*i.e.,* 25/1,471), including at least one from a woman who experienced preterm labor. Twenty-two of these 25 (88%) women had a clinically diagnosed vaginal infection (Table 1), and all but one of these 22 women for whom vaginal pH was recorded exhibited an elevated pH value greater than 4.5 (median pH value = 5.8), an indicator of vaginal dysbiosis. Although microbiome profiles based on 16S rRNA gene surveys are not always accurate measures of the proportions of bacterial taxa present in a sample for a variety of reasons (*e.g.*, biases inherent in DNA extraction, PCR and related sequencing technologies and variations in the number 16S rRNA genes per genome in different species), it is clear that this mycoplasma represents a very abundant taxon in the vaginal samples collected from these 25 women.

**Table 1:** Characteristics of 73 “*Ca*. M. girerdii” positive vaginal samples.

VCU_ID	% “*Ca.* M. girerdii”	qRT-PCR *T. vaginalis*	Current clinical diagnosis	Race/ethnicity	Vaginal pH	*T. vaginalis* genotype	Dominant taxon
VCU_NT02	99.90	+	Trichomoniasis	African American	4.4	ND	“*Ca.* M. girerdii”
VCU_CD82	99.70	+	Trichomoniasis	African American	6	2	“*Ca.* M. girerdii”
VCU_LN42	97.50	+	Trichomoniasis	African American	ND	1	“*Ca.* M. girerdii”
VCU_QM60	94.90	+	Trichomoniasis	African American	5.3	1	“*Ca.* M. girerdii”
VCU_NT41† *	92.90	+	Preterm labor and delivery	African American	6.5	ND	“*Ca.* M. girerdii”
VCU_FQ09	90.60	+	Trichomoniasis	African American	5.5	2	“*Ca.* M. girerdii”
VCU_NT49	90.40	+	None	African American	5.6	ND	“*Ca.* M. girerdii”
VCU_CT62†	89.90	+	Trichomoniasis	Hispanic	6	2	“*Ca.* M. girerdii”
VCU_KH69	88.80	+	Trichomoniasis	Caucasian	7	1	“*Ca.* M. girerdii”
VCU_GK81	88.00	+	Trichomoniasis, Bacterial vaginosis	African American	5.8	AMB	“*Ca.* M. girerdii”
VCU_NT63	87.30	+	Bacterial vaginosis	African American	6	ND	“*Ca.* M. girerdii”
VCU_NT94	85.00	+	Not Available	African American	ND	ND	“*Ca.* M. girerdii”
VCU_AM41	84.20	+	Trichomoniasis	African American	5.8	2	“*Ca.* M. girerdii”
VCU_NT05	82.50	+	Yeast infection	Other (African)	7	ND	“*Ca.* M. girerdii”
VCU_OL06	75.60	+	Trichomoniasis	African American	7	1	“*Ca.* M. girerdii”
VCU_NT03	73.70	+	Bacterial vaginosis	African American	7	ND	“*Ca.* M. girerdii”
VCU_NT54	60.10	+	Trichomoniasis	African American	ND	ND	“*Ca.* M. girerdii”
VCU_MP27	59.60	+	Trichomoniasis	African American	5	1	“*Ca.* M. girerdii”
VCU_NT95	58.80	+	None	African American	7	ND	“*Ca.* M. girerdii”
VCU_BK46	56.10	+	Trichomoniasis	African American	5.5	2	“*Ca.* M. girerdii”
VCU_NT31	51.50	+	Genital warts	African American	5	ND	“*Ca.* M. girerdii”
VCU_NT27	49.70	+	Yeast infection	African American	5.5	ND	“*Ca.* M. girerdii”
VCU_NT44†	48.40	+	Yeast infection	African American	5.5	ND	“*Ca.* M. girerdii”
VCU_NT22	46.10	+	Bacterial vaginosis	African American	5.2	ND	“*Ca.* M. girerdii”
VCU_NT71†	34.90	+	None	African American	5	ND	“*Ca.* M. girerdii”
VCU_RQ48^1^	34.77	+	Trichomoniasis	African American	5.5	2	*Lactobacillus iners*
VCU_NT75	28.15	+	Bacterial vaginosis	African American	ND	ND	NT
VCU_NT96	22.56	+	None	African American	4.5	ND	*Lactobacillus iners*
VCU_QQ25	17.48	+	Trichomoniasis	Other (Biracial)	6.5	1	NT
VCU_NT06	14.89	+	Bacterial vaginosis	African American	5	ND	*Atopobium vaginae*
VCU_NT64	8.63	+	None	African American	4.4	ND	*Lactobacillus iners*
VCU_NT99	8.03	+	None	African American	ND	ND	*Lactobacillus iners*
VCU_VL26	7.90	+	Trichomoniasis	African American	5.8	AMB	*Prevotella*
VCU_NT77^1^	7.51	+	Not Available	African American	6.6	ND	NT
VCU_IV47	7.20	+	Trichomoniasis	African American	5	2	*Gardnerella vaginalis*
VCU_XN28	5.43	+	Trichomoniasis	African American	5	AMB	NT
VCU_NT61	4.09	+	None	African American	ND	ND	*Lactobacillus iners*
VCU_NT50	3.36	+	Bacterial vaginosis	African American	5	ND	*Gardnerella vaginalis*
VCU_QR65*	2.98	+	Trichomoniasis	African American	ND	AMB	*Mycoplasma hominis*
VCU_NT29	2.59	+	Yeast infection	African American	4.5	ND	*Lactobacillus iners*
VCU_NT04	2.21	+	Bacterial vaginosis	African American	5.8	ND	*Atopobium vaginae*
VCU_NT24	2.08	+	Yeast infection	Other (Biracial)	5	ND	*Lactobacillus iners*
VCU_NT55*	1.90	−	None	African American	ND	N/A	*Lactobacillus crispatus*
VCU_SY21	1.76	+	Trichomoniasis	Hispanic	5.5	2	NT
VCU_GF83^3^	1.64	+	Trichomoniasis	African American	5.8	1	*Prevotella*
VCU_NT19	1.59	+	Trichomoniasis	Not available	6	ND	*Gardnerella vaginalis*
VCU_LU24	1.50	+	Trichomoniasis, Bacterial vaginosis	African American	6.1	1	NT
VCU_NT52	1.40	−	Trichomoniasis	African American	5.5	ND	*Gardnerella vaginalis*
VCU_NT69	1.11	+	None (abnormal discharge)	African American	4.5	ND	*Lactobacillus iners*
VCU_BN49	1.06	+	Trichomoniasis, Bacterial vaginosis	African American	5	2	*Gardnerella vaginalis*
VCU_NT68	0.97	+	None	African American	ND	ND	*Lactobacillus iners*
VCU_NT70^2^	0.93	+	None	African American	5.8	ND	*Gardnerella vaginalis*
VCU_NT93	0.92	−	None	African American	5.3	N/A	*Gardnerella vaginalis*
VCU_NT21	0.86	+	Yeast infection	African American	5	ND	*Lactobacillus iners*
VCU_NT60	0.85	+	Trichomoniasis	African American	4.4	ND	BVAB1
VCU_NT67	0.61	+	Yeast infection	Caucasian	4	ND	*Lactobacillus iners*
VCU_NT17	0.59	−	None	Caucasian	4.4	N/A	*Lactobacillus crispatus*
VCU_NT81	0.56	−	None	African American	5.5	N/A	BVAB1
VCU_NT28	0.54	+	None	African American	4	ND	*Lactobacillus iners*
VCU_NT58^2^	0.44	+	None	African American	4.5	ND	*Lactobacillus iners*
VCU_NT09	0.40	+	None	African American	4.6	ND	BVAB1
VCU_QN84	0.39	+	Trichomoniasis	African American	6.1	AMB	BVAB1
VCU_NT59	0.34	−	None	Hispanic	ND	N/A	*Lactobacillus iners*
VCU_NT97	0.29	−	None	African American	ND	N/A	*Lactobacillus crispatus*
VCU_NT40	0.26	+	Bacterial vaginosis	African American	8	ND	*Sneathia amnii*
VCU_NT73^3^	0.20	+	None	African American	5.8	ND	*Lactobacillus iners*
VCU_NT88	0.20	−	None	African American	4	N/A	*Lactobacillus iners*
VCU_XP87	0.15	+	Trichomoniasis, Bacterial vaginosis	African American	ND	2	BVAB1
VCU_NT89	0.13	−	Bacterial vaginosis	African American	4.5	N/A	*Streptococcus agalactiae*
VCU_NT07	0.13	−	None	Hispanic	ND	N/A	*Lactobacillus iners*
VCU_NT26	0.12	+	Bacterial vaginosis	Other (Multiracial)	5	ND	*Gardnerella vaginalis*
VCU_NT62	0.11	ND	Bacterial vaginosis	African American	5.3	N/A	*Gardnerella vaginalis*
VCU_NT91	0.10	−	None	Caucasian	4	N/A	*Lactobacillus iners*

“*Ca*. M. girerdii” was detected in the mid-vaginal microbiome profile at 0.1% or more of total reads. (ND, not determined; AMB, ambiguous; N/A, not applicable; NT, no type).

* Recruited from Labor & Delivery Unit.

† *“Ca.* M. girerdii*”* sequenced genomes VCU-M1, VCU-JB1, VCU-PA1 and VCU-G1 in listed order.

1,2,3 Three “*Ca*. M. girerdii” positive samples were from women who enrolled in the study more than once. VCU_GF83 was collected 424 days after VCU_NT73; VCU_NT58 was collected 246 days after VCU_NT70; VCU_NT77 was collected 117 days after VCU_RQ48.

We examined the association between vaginal carriage of the novel bacterium, even as a minor component of the microbiome, and common clinically diagnosed vaginal infections. The association between “*Ca*. M. girerdii” and trichomoniasis was highest of several vaginal organisms of the female urogenital tract with a relative risk of 20.12 (Table 2). *M. hominis*, which has previously been linked with trichomoniasis [9], [10], exhibits a much weaker association with a relative risk of 2.53, likely at least in part due to its strong association with BV. We did not find “*Ca*. M. girerdii” to be associated with an elevated relative risk for BV as diagnosed by Amsel’s criteria [19]. Amsel’s criterion assessment provides a dichotomous test with a relatively high specificity, but relatively low sensitivity [20]. BV assessed the Nugent’s Gram-stain criteria [21] represents the continuum of alterations in vaginal flora. Both pregnant [22] and non-pregnant [2] women with intermediate Nugent scores have been reported to be more likely to have trichomoniasis. While Nugent scores were not recorded in this study, the 16S rRNA microbiome profiles (Figure S1) are consistent with the hypothesis that women co-infected with “*Ca*. M. girerdii” and *T. vaginalis* may also be more likely to have intermediate flora.

**Table 2:** Associations of vaginal carriage of bacterial taxa with common vaginal infections.

	Relative Risk (95% Confidence Interval)		
	Trichomoniasis	Bacterial vaginosis	Yeast infection
“*Ca*. M girerdii”	20.12 (7.75–48.34)	0.88 (0.24–1.53)	0.86 (0.00–1.98)
*M. hominis*	2.53 (0.85–6.83)	2.08 (1.61–2.68)	0.80 (0.44–1.30)
*U. parvum/U. urealyticum*	1.36 (0.40–3.49)	0.62 (0.45–0.84)	1.18 (0.74–1.75)
*Gardnerella vaginalis*	4.45 (0.91– Infinity*)	7.17 (4.05–21.78)	0.81 (0.54–1.32)
*Atopobium vaginae*	1.79 (0.70–9.10)	5.02 (3.50–8.43)	0.56 (0.35–0.83)
BVAB2	0.66 (0.17–1.80)	3.25 (2.54–4.30)	0.45 (0.22–0.75)

Bootstrap (n = 1,000) samples were selected from the outpatient clinic population to reflect the outpatient community composition. Median relative risk and 95% bootstrap confidence intervals are shown. A bacterial taxon was considered present in the mid-vaginal sample if at least 0.1% of the metagenomic 16S rRNA gene microbiome profile reads classified to the taxon. Vaginal infection was determined by clinical diagnosis using Amsel’s criteria for BV.

*For at least 2.5% of the bootstrap samples, all subjects with a trichomoniasis diagnosis were positive for *G. vaginalis*.

We detected “*Ca*. M. girerdii” at threshold of at least 0.1% of the 16S profile in 28 of the 63 (44.4%) women with clinically diagnosed trichomoniasis. We also found the new mollicute at less than 0.1% of the 16S rRNA threshold in eight additional women with trichomoniasis. Thus, we were unable to detect “*Ca.* M. girerdii” in the 16S rRNA gene profiles of only 27 of the 63 (42.9%) women with clinically defined trichomoniasis. In this study, trichomoniasis was clinically diagnosed by wet prep microscopy rather than culture and microbiome profiles were generated using the V1-V3 hypervariable region of the 16S rRNA gene rather than the V4-V6 region used by others [2]. Despite these methodological differences and differences in the study populations, we confirmed a strong association between the presence of *T. vaginalis* and “*Ca*. M. girerdii” previously reported as statistically significant (p = 0.026) by Martin *et al.* [2].

### Association of “Ca. *M. girerdii*” with *T. vaginalis*

**Figure 1:**
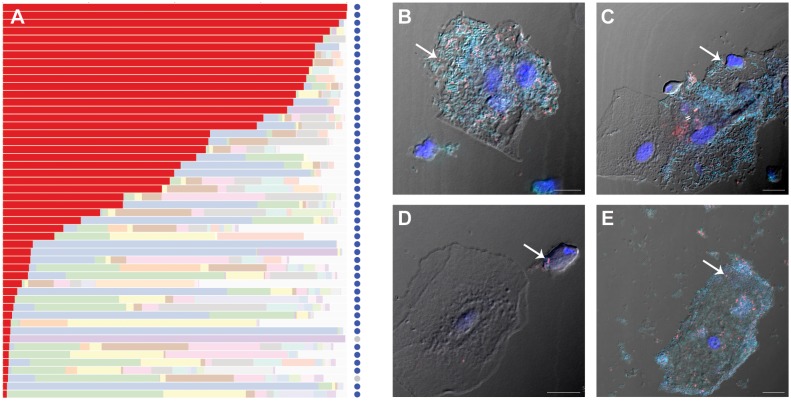
Detection of “*Ca*. Mycoplasma girerdii” in mid-vaginal samples. Panel (A) shows relative abundance of major taxonomic groups in “*Ca*. M. girerdii” positive samples (1% 16S rRNA threshold): “*Ca*. M. girerdii” is colored red (A). Light colored bars represent other taxa. Dark blue circles represent samples positive for *T. vaginalis* by qRT-PCR, light gray circles represent negative samples. Panels (B–E) show fluorescence *in situ* hybridization detection of bacteria in mid-vaginal samples from two participants with clinically diagnosed trichomoniasis (subject 1, panels B, C and D; subject 2, panel E) by confocal laser scanning microscopy. Most bacteria were detected with fluorescein-labeled broad-range bacteria probe Eub338 (turquoise). “*Ca*. M. girerdii” was also stained with a Cy5-labeled probe targeting 16S rDNA (red). Nuclei were labeled with 4′6′-diamidine-2-phenylindole, dehydrochloride (DAPI, blue). Negative control with reverse complementary probe of Eub338 did not hybridize to any bacteria (data not shown). Scale bar = 10 µm.

Up to half of all *T. vaginalis* infections are asymptomatic and undiagnosed [11]. We performed real-time qRT-PCR on all mid-vaginal samples positive for “*Ca*. M. girerdii” and found that 49 of the 51 (96%) women who carried the mycoplasma at a 1% threshold by 16S rRNA gene profiling also carry *T. vaginalis* (Figure 1A; Table 1). Even at a lower 16S rRNA threshold of 0.1%, 61 of 72 (85%) of women who carried “*Ca*. M. girerdii” were *T. vaginalis* positive. Thus, “*Ca.* M. girerdii” exhibits an unusually strong correlation with trichomoniasis. We also found that “*Ca*. M. girerdii” was associated with both of the previously described genotypes of *T. vaginalis* [23], [24], type 1 and type 2 (Figure S3), indicating a broad-range association with this infectious disease. Both *T. vaginalis* genotypes have been reported in the HIV-positive women [25]. Additional studies are needed to determine whether “*Ca*. M. girerdii” co-infection contributes to the increased risk of HIV acquisition and transmission or to adverse pregnancy outcomes associated with trichomoniasis.

**Figure 2:**
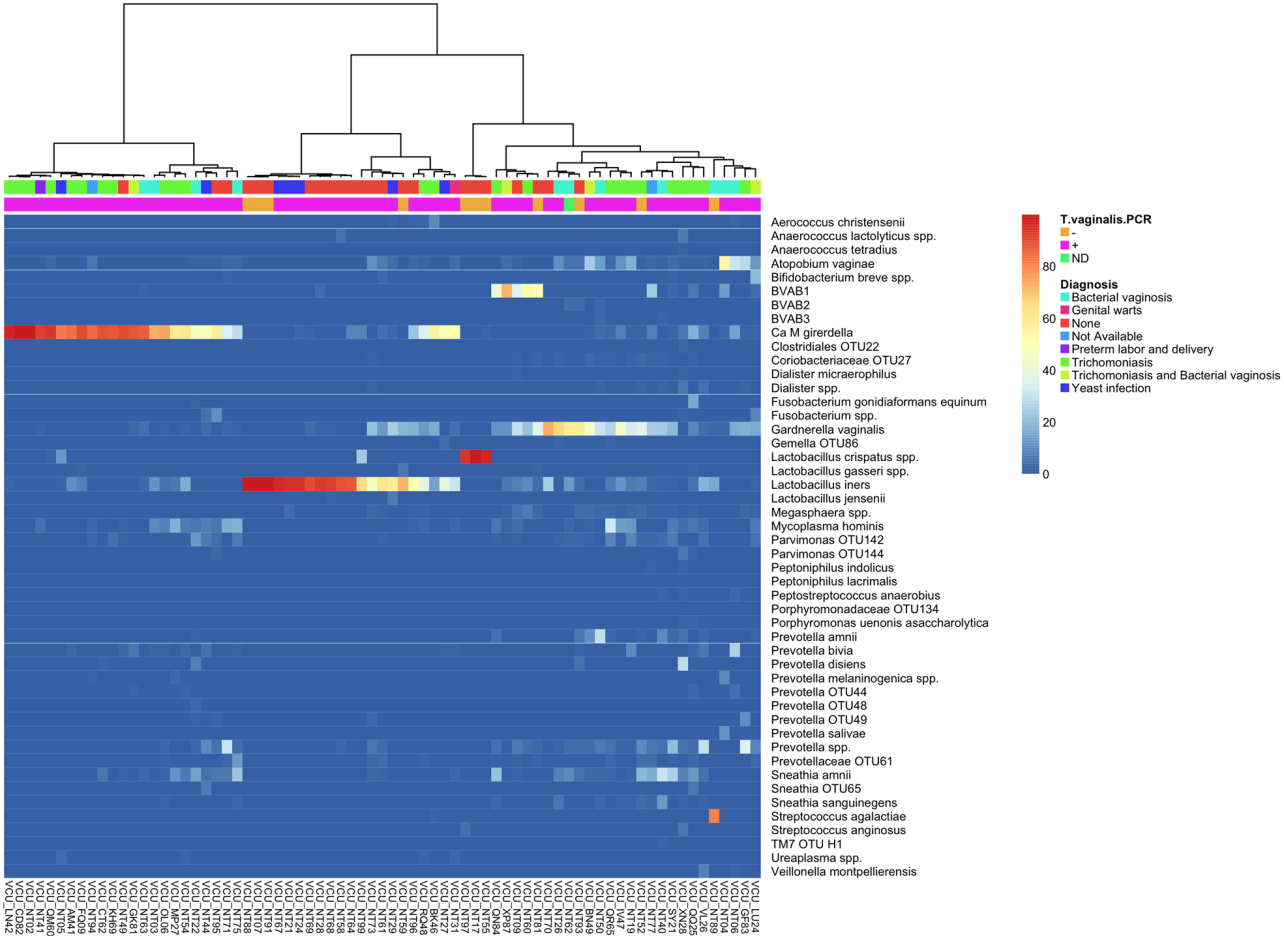
Cluster analysis of mid-vaginal samples positive for “*Ca*. M. girerdii”. Relative abundance of microbial taxa in mid-vaginal bacterial communities of “*Ca.* M. girerdii” positive women is shown. The dendrogram was generated using Ward’s method with Manhattan distance. This analysis includes only mid-vaginal samples that exhibited at least 0.1% “*Ca*. M. girerdii” by 16S rDNA profiling. Clinical diagnosis is indicated in the first bar, and presence of *T. vaginalis* by RT-PCR is indicated in the second bar (orange designates a negative result and pink designates a positive result). The three samples dominated by *L. crispatus* and the three samples with the highest prevalence of *L. iners* were negative for *T. vaginalis*.

Interestingly, of 22 women with no diagnosis who were positive for “*Ca*. M. girerdii”, 14 were also positive for *T. vaginalis* (Table 1). *Lactobacillus crispatus* is associated with decreased rates of *T. vaginalis* infection [12], and we found that the three “*Ca*. M. girerdii” positive women with a predominance of *L. crispatus* were negative for *T. vaginalis* (Figures 2 and S1). Thus, although our data are supportive of a dependent relationship, it appears that “*Ca*. M. girerdii” may not absolutely require *T. vaginalis* to colonize the human vagina. Our data suggest vaginal carriage of the new mycoplasma is associated with elevated vaginal pH and African American race (Table 3), risk factors for preterm birth [26], which are also associated with BV [27] and trichomoniasis [28]. Given the tight association of the mycoplasma with *T. vaginalis*, it is not possible to determine whether the organism is independently associated with these factors.

**Table 3:** “*Ca*. M. girerdii” is associated with African American race and elevated vaginal pH.

	African American race	Caucasian race	Mid-vaginal pH
“*Ca*. M. girerdii”	88.0/74.8(0.003*)	6.9/17.6(1)	5.5/5.0(0.006*)
*Mycoplasma hominis*	89.4/70.1(0*)	6.2/21.2(1)	5.5/5.0(0*)
*Ureaplasma spp.*	75.1/75.3(0.555)	17.0/17.2(0.571)	5.0/5.0(0.514)
*Gardnerella vaginalis*	81.5/59.2(0*)	11.9/30.8(1)	5.3/4.6(0*)
*Atopobium vaginae*	85.6/62.2(0*)	8.8/27.6(1)	5.3/4.6(0*)
BVAB2	88.1/69.0(0*)	6.5/22.3(1)	5.5/5.0(0*)

Mean proportions of the most common racial groups and median values for mid-vaginal pH were calculated for carriers and non-carriers (*i.e.,* 0.1% 16S rRNA microbiome threshold) of the genital mycoplasmas and representative BV-associated species of women recruited from 1,361 women recruited from outpatient clinics. Bootstrap analysis was performed so that values represent the underlying population of the clinic.

*Bootstrap probabilities (n = 1,000) less than 0.05 indicate that the mean proportion or median value is significantly higher for carriers of the species.

Fluorescence *in situ* hybridization (FISH) on vaginal samples with a representation of “*Ca*. M. girerdii” (Figure 1B–E, Figure S2) showed that the bacterium is prominent in polymicrobial biofilms sometimes associated with “clue cells” (Figure 1B, 1C, 1E), a characteristic of BV. The mycoplasma was also dispersed with other bacteria and only occasionally co-localized with *T. vaginalis* (Figure 1D). It is not yet clear whether “*Ca*. M. girerdii” can enter and replicate inside of *T. vaginalis* like *M. hominis* [29], penetrate human cells like *M. penetrans* [30], or whether the mycoplasma is strictly extracellular. Given that eight women carrying the mycoplasma were negative for *T. vaginalis* (Table 1), our data suggest “*Ca.* M. girerdii” is not an obligate symbiont of the parasite as suggested by Martin *et al.* [2]. Symbiotically-associated *M. hominis* and *T. vaginalis* have recently been shown to synergistically upregulate the proinflammatory response [31]. Further studies are required to determine whether a symbiotic relationship between “*Ca*. M. girerdii” and *T. vaginalis* may similarly synergize to influence host response.

### Genomic and Phylogenetic Analyses of “*Ca*. M. girerdii”

**Figure 3:**
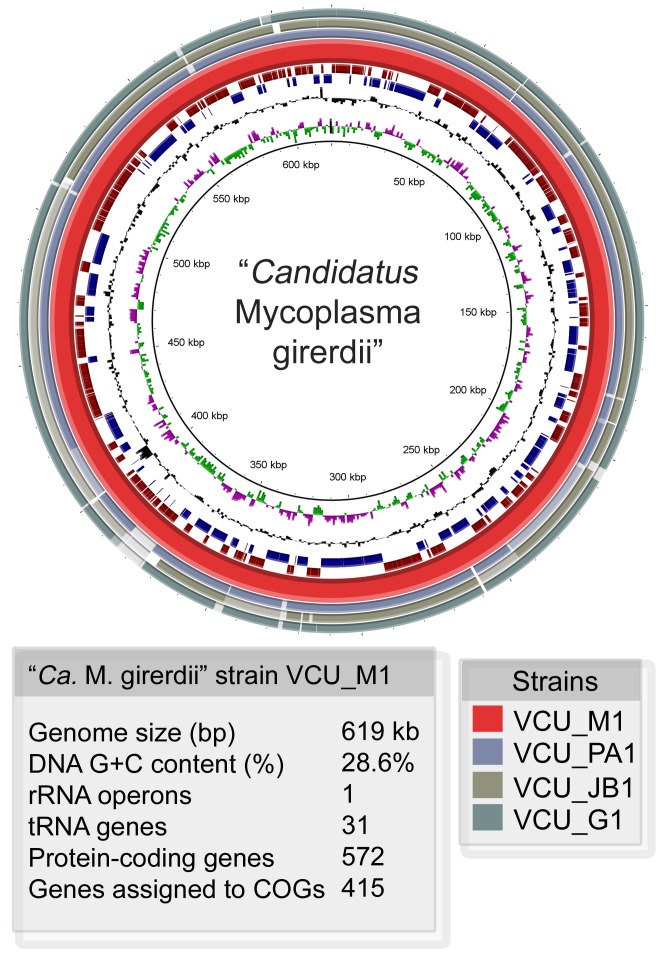
Representation of “*Ca.* M. girerdii” genomes. A circular representation of the “*Ca.* M. girerdii” reference genome (strain VCU_M1) assembled from metagenomic sequences from a mid-vaginal sample. Position 1 is set to the start of the *dnaA* gene. Outermost circles (1–3) show the alignment (97% or greater identity) of contigs of three different strains from metagenomic assemblies from mid-vaginal samples containing high proportions of “*Ca*. M. girerdii”. Circle 4 (red) represents the reference strain (VCU_M1). Circles 5 (dark red) and 6 (blue) represent the predicted coding sequences in the forward and reverse orientations respectively. Circle 7 (black) shows the GC content, and circle 8 shows GC skew (pink (-), green (+)).

Our attempts to cultivate “*Ca*. M. girerdii” have not succeeded. Therefore, we employed the strategy of assembling whole metagenome shotgun sequence reads to complete the genome of a reference strain of this organism. The ∼619 kb genome is ∼28.6% GC content and features sequences for ∼572 putative proteins, 34 structural RNAs and one predicted CRISPR locus (Figure 3). Three additional strains from other samples were similarly assembled and aligned to the reference. Gene synteny was very high among the four strains, but limited with other related species (Figure S4). The four strains of “*Ca*. M. girerdii” exhibited an average of 99.8% nucleotide identity.

The reference genome exhibits irregular GC skews with no distinctive inversion (Figure 3), which is indicative of the high genome plasticity that is typical for mycoplasmas and consistent with the overall lack of synteny with related species. In the reference strain, one putative CRISPR locus containing four 34-nucleotide repeats and three 35-nucleotide spacers was identified in the genome with a consensus direct repeat sequence of 5′-AAGTATTAATATTCCAAGTAGTGTAACTAGTATT. Genes in the rRNA operon were organized 5′-16S-23S-5S, and no tRNAs were identified in the intergenic transcribed spacer regions as with most *Mycoplasma* and *Ureaplasma* species [32]. Like other mycoplasmas, “*Ca*. M. girerdii” possesses a minimal number of tRNAs and utilizes UGA as a tryptophan codon. We identified 31 putative tRNAs: 11 amino acids represented by a single anticodon; seven amino acids (Gly, Lys, Ser, Thr, Trp, Met) represented by two anticodons; and two amino acids (Leu, Arg) represented by three anticodons. Some, but not all, mycoplasmas have lost the tRNA-Trp gene that utilizes the TGG codon[33], but “*Ca*. M. girerdii” appears to have both. We identified two tRNA-Trp genes, one that utilizes the UGA codon with an observed codon frequency of ∼0.87% and another that utilizes the TGG codon with an observed codon frequency of ∼0.13%. The *dnaA* and *dnaN* genes are co-localized, but *recF* appears to be absent and *gyrB* is only distantly linked. As with *M. penetrans* and *U. urealyticum* [34], no clusters of DnaA boxes were identified upstream of the dnaA gene, as only one 9-mer with two base differences from the DnaA box consensus (5′-TTATCCACA) was identified in that region.

Homologs to putative virulence factors, including collagenases, a hemolysin, an O-sialoglycoprotein endopeptidase, and a *feoB*-like iron transport system, were identified in all four strains. Intriguingly, tensile strength of fetal membranes is imparted by collagens, and thus bacterial collagenase activity could facilitate fetal membrane rupture. “*Ca*. M. girerdii” appears to lack the superoxide dismutase gene, but encodes a complete desulfoferrodoxin-type superoxide reductase system that likely functions to protect against oxidative stress. A ∼16 kb plasmid is apparently present at approximately two copies per “*Ca*. M. girerdii” cell in the sample containing the reference strain, but was not observed in the samples containing the other strains. It carries a plasmid replication initiator protein, two genes resembling components of a type IV secretion system and ∼9 hypothetical genes. Because of its prevalence in the former sample and its similarity to plasmids from related organisms, it is possible that this may represent the first plasmid associated with a mycoplasma in this phylogenetic group (see below).

**Figure 4:**
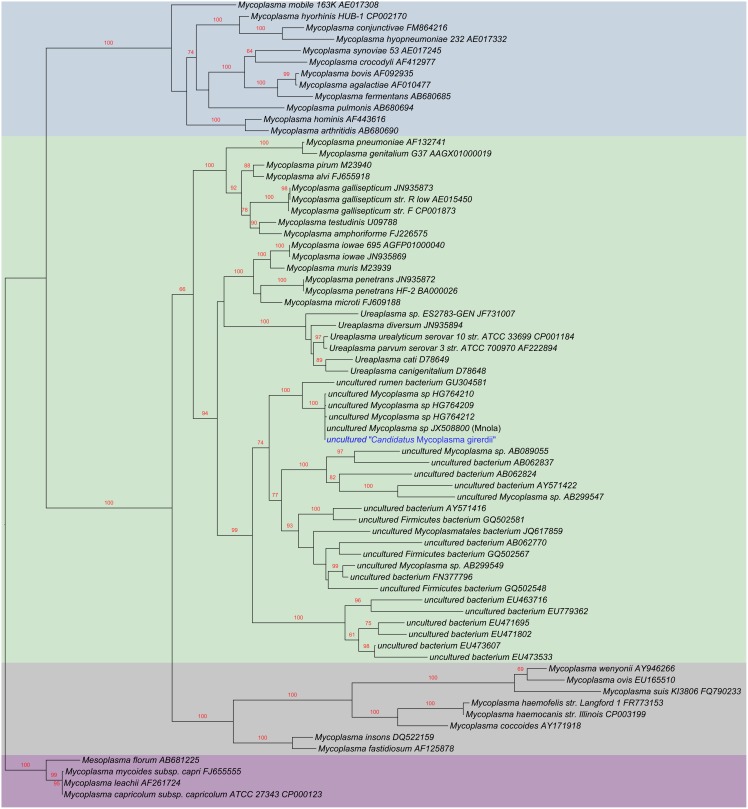
Phylogenetic tree of 16SrRNA shows uncultured “*Ca.* M. girerdii” clusters most closely with other uncultivated organisms in the Pneumoniae Group. The maximum likelihood tree was inferred by RAxML 7.2.7 using the gamma-distributed heterogeneity rate categories with 1,000 bootstraps. The 16S rRNA gene alignments were manually inspected. The Hominis Group is shaded in blue, the Pneumoniae Group in green, the Hemoplasma Group in gray and the Spiroplasma Group in purple. The 16S rRNA sequence of “*Ca*. M. girerdii” VCU_M1, “*Ca*. M. girerdii” VCU_PA1, “*Ca*. M. girerdii” VCU JB1 and “*Ca*. M. girerdii” VCU_G1 were identical. “*Ca*. M. girerdii” groups most closely with “Mnola”, which shows 100% identity in 16S rRNA sequence, uncultivated organisms from the oral sample of a low birth weight infant (HG764209, HG764210, and HG764212) and uncultivated species from rumen and termite gut in the Pneumoniae Group. A partial 16S rRNA sequence from the vaginal sample of a woman who delivered full term (JX871253) also exhibits 99% identity with “*Ca*. M. girerdii”, but was not included in the analysis due to its length.

Phylogenetic analysis of 16S rRNA genes shows that “*Ca.* M. girerdii” is most closely related to other uncultivated organisms identified by 16S rRNA sequence: the organism reported by Martin *et al*. [2], an organism identified by Costello *et al*. in oral samples of a low birth weight neonate [16], and other organisms from bovine rumen [35], the gut of termites [36]–[38] and Asiatic elephant and Somali wild ass feces [39] (Figure 4). Interestingly, the environments of the gut of lower termites, the rumen of cattle and other foregut fermenters and the cecum of hindgut fermenters (*e.g*., Asiatic elephant and Somali wild ass) are all models of symbiosis where diverse groups of organisms including bacteria and protozoa contribute to carbohydrate fermentation and benefit the host by assisting with plant digestion.

**Figure 5:**
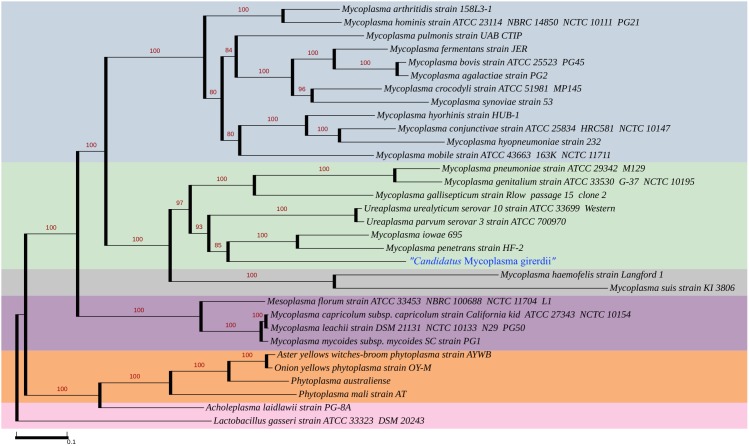
Phylogenetic Tree based on inferred amino acid sequences confirms placement of “*Ca.* M. girerdii” in the Pneumoniae group. “*Ca*. M. girerdii” is located within the Pneumoniae group, denoted in green, in a subclade along with the *Ureaplasma* species, *M. iowae* and *M. penetrans*. The tree was inferred using amino acid sequences of 57 orthologs (Tables S4, S5, and S6). Numbers at nodes correspond to the support values from 1,000 bootstrap replicates.

A phylogenetic analysis using 57 inferred orthologous proteins placed “*Ca*. M. girerdii” in the Pneumoniae group with *M. penetrans* and *Mycoplasma iowae*, relatively distant from *M. hominis* (Figure 5). This phylogeny is supported by analysis. Analysis of COGs distributed 415 of the “*Ca.* M. girerdii” genes among functional categories in a pattern similar to that exhibited by other mycoplasma species (Table S1). The full-length “*Ca*. M. girerdii” 16S rRNA gene from all four genomes and “Mnola” exhibited 100% identity.

### Metabolic strategies of “*Ca*. M. girerdii”

**Figure 6:**
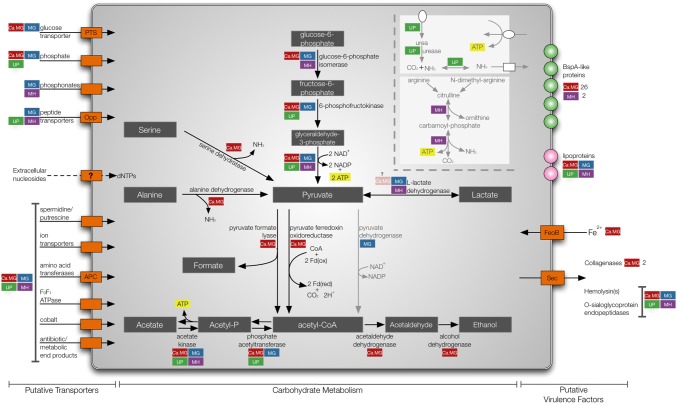
Unique strategies of “*Ca*. M. girerdii”. Putative transporters, enzymes involved in carbohydrate metabolism and virulence factors are represented in red for “*Ca*. M. girerdii”. Comparisons with other genital mycoplasmas are indicated with color-coded boxes: *M. genitalium* (MG, blue) *U. parvum* (UP, green) and *M. hominis* (MH, purple). Arrows indicate direction of transport. Light gray arrows represent metabolic strategies unique to other genital mycoplasma. Metabolic reconstruction was performed using ASGARD and careful inspection of manual annotations.

Genital mycoplasmas have evolved to utilize various energy sources; *M. genitalium*, *M. hominis*, and ureaplasmas use glucose, arginine and urea respectively. Our metabolic reconstructions suggest that “*Ca*. M. girerdii” is glycolytic like *M. genitalium* and encodes all enzymes for utilization of glucose as an energy source (Figure 6). Arginine dihydrolase pathway and urease genes are absent, thus “*Ca*. M. girerdii” is not predicted to utilize arginine and urea. Catabolism of galactose, mannose, sucrose, maltose, glycogen, starch or glycerol is not predicted, and the roles of genes in the lactose/galatose pathways are unclear. “*Ca*. M. girerdii” possesses genes for a putative IIA component of the lactose-specific phosphotransferase system (MGM1_4770), ribose/galactose-ABC-type transporter system (MGM1_3070, MGM13080) and a galactose-6-phosphate isomerase (MGM1_4760/MGM1_4750). However, other genes required for lactose and galactose catabolism, including 6-phospho-beta-galactosidase, tagatose-6-phosphate kinase and tagatose-bisphosphate aldolase, were not identified. Moreover, the L-lactate dehydrogenase gene (MGM1_4130) has an apparent frameshift. Other components of the phosphotransferase system (PTS) system were also identified, including the HPr phosphocarrier protein (MGM1_1420) and an HPr phosophatase/kinase (MGM1_4210), which likely functions in the regulation of carbon metabolism. While all eight subunits for the F1F0 ATPase complex were identified (MGM1_4310 through MGM1_4380), these genes are thought to be involved in maintenance of the proton gradient rather than ATP generation in mycoplasma species as the cytochrome components are absent.

Unique among the genital mycoplasmas, the “*Ca*. M. girerdii” genome encodes serine dehydratase (MGM1_2560, MGM1_0390), alanine dehydrogenase (MGM1_5480, MGM1_1820), and 2′,3′-cyclic-nucleotide 2′-phosphodiesterase (MGM1_1930) that may permit use of alternate energy sources in the absence of glucose: L-alanine, L-serine, and 2′,3′ cyclic AMP. No serine dehydratases have been previously described for the mollicutes, and while alanine dehydrogenase has been described for *Acholeplasma laidlawii* [40] and annotated for *M. mycoides* (ADH22225.1), *M. mobile* (AAT27586.1), *M. leachii* (ADR24467.1), and *M. putrefaciens* (YP_004790384.1), neither the gene nor the enzyme has been previously identified in any of the genital mycoplasmas or organisms classified in the Pneumoniae group. Moreover, 2′,3′-Cyclic phosphodiesters may be available in the environment as intermediate products in the hydrolysis of RNA by ribonuclease I. This strategy has been proposed for *Yersinia enterocolitica* [41], which has been shown to grow on 2′,3′-cAMP as a sole carbon source. “*Ca*. M. girerdii” does not metabolize pyruvate through the pyruvate dehydrogenase pathway that is used by *M. genitalium* or the other mycoplasma species that catabolize pyruvate to acetate. However, “*Ca*. M. girerdii” may utilize one or both of two alternate enzymes identified in the genome: pyruvate-formate lyase (MGM1_5430), which produces acetyl-CoA and formate from CoA and pyruvate, and/or pyruvate ferredoxin/flavodoxin oxidoreductase (MGM1_5310), which yields acetyl-CoA and carbon dioxide from the same substrates by reducing either ferredoxin or flavodoxin. Both of these enzymes seem to be unique to “*Ca*. M. girerdii” among the mycoplasmas. Acetyl-CoA may be converted to acetate by phosphate acetyltransferase (MGM1_0120) and acetate kinase (MGM1_2290), resulting in the production of ATP.

As with other mollicutes, “*Ca*. Mycoplasma girerdii” appears to have limited metabolic capabilities and imports much of what it needs from its environment or host. “*Ca*. M. girerdii” seems to lack gluconeogenesis and the TCA cycle like other mycoplasmas. It lacks enzymes for *de novo* purine or pyrimidine synthesis and amino acid synthesis, but appears to be capable of nucleotide salvage and amino acid transport. The genome encodes ∼40 genes associated with transport of various ions and substrates including amino acids, glucose, ribose/lactose, potassium ion, magnesium ion, calcium ion, ferrous iron, cobalt, phosphate and spermidine/putrescine (Table S2). An alcohol dehydrogenase (MGM1_5890) exhibiting homology to a butanol dehydrogenase and putative bifunctional aldehyde-alcohol dehydrogenase (MGM1_1150) were also annotated in the genome, thus ethanol may also be an end product of metabolism. Although “*Ca*. M. girerdii” is predicted to be able to convert butanol to butanoyl-CoA, it appears to lack other enzymes of butanoate metabolism. The predicted metabolic reconstruction may provide insight and help guide future cultivation attempts.

### BspA-like proteins encoded in “*Ca*. M. girerdii”

**Figure 7:**
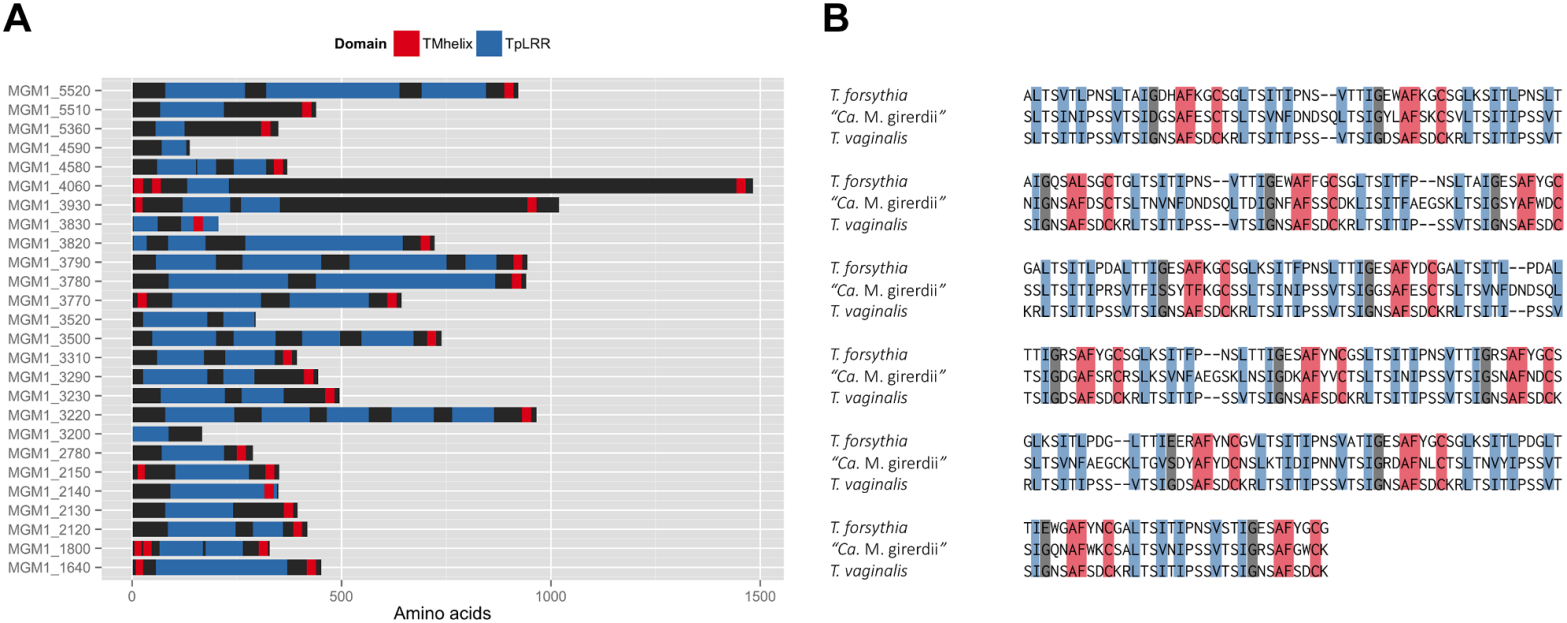
Expanded “*Ca*. M. girerdii” BspA-like protein family. The diverse family of 26 BspA-like protein family members from the “*Ca*. M. girerdii” strain VCU_M1 is depicted in panel (A). Predicted transmembrane domains and TpLRR domains are represented. An alignment of TpLRR domains from *Tannerella forsythia* BspA (AAC82625.1, bases 382–1347), “*Ca*. M. girerdii” strain VCU_M1 MGM1_3780 (bases 449–782) and *T.vaginalis* BspA-like TVAG_495790 (XP_001327783.1, bases 112–1077) is shown in panel (B).

*Mycoplasma* species contain surface proteins that exhibit high frequency antigenic variation [42]. Although these organisms exhibit a low level of horizontal gene transfer, expanded families of surface proteins are an exception [43]–[45]. In the reference genome of “*Ca.* M. girerdii”, we identified a family of 26 BspA-like proteins containing *Treponema pallidum* leucine rich repeat (TpLRR) domains with homology to the prototypical BspA virulence factor of *Tannerella forsythia* and a family of over 900 BspA-like proteins of *T. vaginalis* [46], [47]. BspA-like proteins from “*Ca*. M. girerdii” exhibit variable length ranging in length from 136 to 1481 amino acids (Figure 7). Twenty-three of the BspA-like proteins contained a predicted C-terminal transmembrane domain, and a signal peptide was detected for five of the BspA-like proteins.

Members of this family stimulate a Toll-like receptor 2 (TLR2)-mediated host immune response. We also identified two other putative surface lipoproteins that lack the TpLRR domain, but exhibit homology to other mycoplasma proteins that stimulate a TLR-mediated innate immune response. It is intriguing to hypothesize that the expanded families of BspA-like proteins in the “*Ca*. M. girerdii” and *T. vaginalis* may represent a common host-adaptation strategy.

The BspA from *T. forsythia* is perhaps the best studied protein containing the TpLRR domain. This protein has been shown to mediate a host innate immune response through Toll-like receptor 2 (TLR2 [48]) by directly interacting with the receptor [49]. More recently, the protein has been shown to elicit a response through scavenger receptor gp340 [50]. *T. forsythia* BspA has also been shown to be required for host cell attachment and invasion [51] and co-aggregation with *Fusobacterium nucleatum* [51]. Thus, the “*Ca*. M. girerdii” BspA-like proteins may also mediate interactions with the host and contribute to virulence through induced host inflammation.

No TpLRR-containing proteins have been identified in *U. parvum*, *U. urealyticum* or *M. genitalium* and only one or two predicted BspA-like proteins were identified in the other related genomes examined: one in *M. iowae* (ZP_08916569.1), one in *M. fermentans* (YP_003922737.1 and YP_003922552.1), two in *M. hominis* (YP_003302763.1; YP_003303010.1) and one in *M. penetrans* (NP_757414.1). Although the role of the 26 member BspA-like gene family in “Ca. M. girerdii” is currently unclear, it is likely that they have important functions in host interactions. It is intriguing to hypothesize that the TpLRR domains of the BspA-like proteins from *M. hominis*, *T. vaginalis*, and “*Ca*. M. girerdii”, which appear to be absent in the Ureaplasma species and *M. genitalium* genomes, may interact with the human host cells in a similar manner via the TLR2 receptor.

## Conclusions

In summary, we confirmed the identity of a recently described and still uncultivated species of mycoplasma, further documented its strong association with the presence of *T. vaginalis*, and comprehensively characterized the genomes of “*Ca*. M. girerdii” from four vaginal samples collected in the Vaginal Human Microbiome Project at VCU. The genomes of this potentially emerging pathogen provide insight into its metabolic strategies and reveal a potential for virulence and for triggering host inflammatory responses through innate immune mechanisms. This work lays the foundation for understanding the impact of “*Ca.* M. girerdii” on women’s urogenital health and the nature of its association with *T. vaginalis*.

### Description of “*Candidatus* Mycoplasma girerdii”

“*Candidatus* Mycoplasma girerdii” [gir.erdii. N.L. gen. n. *girerdii*, named for P.H. Girerd, an American obstetrician and gynecologist, for his dedication to clinical practice and his contributions to the research of the vaginal microbiota].

## Materials and Methods

### Identification of “*Ca*. M. girerdii”

Mid-vaginal swab samples collected from women at 1,471 visits (1,361 outpatient visits, 110 visits to the labor and delivery unit) were assayed by 16S rRNA gene pyrosequencing according to the protocols of the Vaginal Human Microbiome Project at VCU [18], [52]. The team that performed the PCR was blinded to the BV and *T. vaginalis* diagnoses and results. Mid-vaginal pH, clinical diagnosis and health history were recorded. The teams that recorded and digitally entered this information were blinded to the PCR and RT-PCR results. Clinical diagnosis of trichomoniasis was based on identification of motile trichomonads in a saline wet mount preparation of vaginal discharge. BV was clinically diagnosed in women meeting at least three of Amsel’s four criteria [19]: characteristic BV discharge, clue cells on microscopy, vaginal pH >4.5 and positive whiff test. Consent was obtained from all participants in accordance with the study protocol (HM12169) as approved by the institutional review boards for human subjects protection at Virginia Commonwealth University and the Virginia Department of Health Raw sequence data from the project is available from the Short Read Archive at NCBI (projectID phs000256).

### Fluorescence *in situ* hybridization

Vaginal swabs were resuspended in phosphate buffered saline (PBS) and incubated on poly L-lysine coverslips (BD Biosciences, San Jose, CA) in a 24-well tissue culture plate for 30 min at 37°C. Coverslips were washed with PBS, fixed in 4% paraformaldehyde/PBS for 1 h, and permeabilized with 0.2% TritonX-100/PBS for 10 min. Fifty nanograms of fluorescently labeled probe targeting bacterial 16S rRNA was added to 200 µl of pre-warmed hybridization buffer (0.9 M NaCl, 20 mM Tris-HCl (pH 7.2), 10% formamide, 0.01% SDS). Hybridization was carried out at 45°C for at least 2 h. Fluorescein-labeled broad range bacteria probe Eub338 (5′GCTGCCTCCCGTAGGAGT-3′) was used as a control. A Cy5-labeled specific probe (5′-TCCTCTTAGTGCCGTTCGTCC-3′) was used to detect “*Ca*. M. girerdii”. Following hybridization, coverslips were rinsed with pre-warmed hybridization wash buffer (0.45 M NaCl, 20 mM Tris-HCl (pH 7.2), 0.01% SDS) then incubated in the wash buffer for 15 min at 48°C. Immediately following, coverslips were washed with ice-cold distilled H_2_O, dried for 15 min at 48°C and mounted with ProLong Gold Antifade containing 4′,6-Diamidino-2-phenylindole (DAPI) (Invitrogen, Carlsbad, CA). Slides were visualized by laser scanning confocal microscopy using a Zeiss LSM 700.

### Detection and genotyping of *T. vaginalis*

Detection of *T. vaginalis* by quantitative real-time RT-PCR was performed as described by Shirm *et al.* [53], and *T. vaginalis* genotyping was performed using three single-copy genes as described by Conrad *et al.* [23], [24].

### Relative Risk Analysis

Bootstrap (n = 1,000) samples were selected from the outpatient clinic population to reflect the outpatient community composition. Samples from women enrolled in labor and delivery were not included in this analysis. Median relative risk and 95% bootstrap confidence intervals were calculated. A bacterial taxon was considered present in the mid-vaginal sample if at least 0.1% of the metagenomic 16S rRNA gene microbiome profile reads classified to the taxon.

### Metagenomic assembly of “*Ca*. M. girerdii”

We selected one mid-vaginal sample (VCU_NT41; Table 1) with >90% of 16S rRNA reads classified to “*Ca*. Mycoplasma girerdii” from which to assemble the reference genome of the organism. The woman who provided this sample was in active preterm labor and also tested positive for group B Streptococcus (GBS), *Chlamydia trachomatis* and *T. vaginalis*.

Fifty nanograms of total DNA was used in a tagmentation reaction with a Nextera DNA Sample Prep Kit (Roche Titanium-compatible, Epicentre Biotechnologies) following the manufacturer’s protocol and sequenced in the Nucleic Acids Research Facilities at VCU. Titanium FLX pyrosequencing (Roche/454; 1/2 plate) yielded 793,732 reads and 241,486,162 bases. The raw data was pre-filtered to remove most human reads (55% of the total reads) using Bowtie 2 [54] with default parameters. The reads were then split into two bins using AbundanceBin [55]: (1) a bin containing abundant reads including those derived from “*Ca*. M. girerdii”, and (2) a bin containing less abundant reads derived from the minor components of the vaginal microbiome. Human-filtered reads from the bin of abundant sequences (*i.e.*, 252,073 reads, 79,029,547 bases) were assembled using Newbler, resulting in 298 contigs larger than 500 bases and 1,966 contigs larger than 100 bases, with a total of 89.10% of reads aligned to a contig. Through a careful analysis of the single-end read flow information from Newbler, we inferred a circular scaffold for the organism that incorporated 19% (152,023 total reads) of the total unfiltered reads from the metagenomic sample. The scaffold incorporated eight of the largest ten contigs that ranged in size from 144,547 bases to 4,826 bases and exhibited 67.2-fold to 95-fold coverage. Sequence reads incorporated into the “*Ca*. M. girerdii” genome did not map to known *Mycoplasma* or *Ureaplasma* sequences, and no other unnamed mollicutes were detected in these samples by 16S rRNA analysis. The eighth-largest contig encoded a 16kb plasmid with an observed depth of 189-fold coverage. Because of its abundance, it is likely that this plasmid is from “*Ca*. M. girerdii” but the host of the plasmid cannot yet been unequivocally assigned. The ninth-largest contig aligned to *T. vaginalis* ribosomal RNA genes. An additional eight smaller contigs ranging in size from 161 bases to 814 bases were also incorporated into the scaffold, with two of the contigs incorporated twice. The majority of non-assembled contigs aligned to *T. vaginalis* (232 contigs), *Homo sapiens* (21 contigs), or other bacterial species; *e.g., Gardnerella vaginalis* (10 contigs). Physical gaps in the contig junctions were confirmed and closed by PCR across gaps and fluorescent chain termination sequence analysis on the AB3730 or AB3130 capillary sequencers (Applied Biosystems). These gaps commonly occurred either in genes exhibiting homology to type I restriction modification system proteins or those encoding BspA-like proteins, although one junction spanned a gene encoding a signal recognition particle protein, which was present in only one copy. PCR and sequencing primers are provided (Table S3). All physical gaps in the scaffold were closed by PCR-amplification and sequencing using the Sanger capillary methodology. The circularity of the 16kb plasmid was similarly confirmed.

Three additional mid-vaginal samples (VCU_CT62, VCU_NT44, VCU_NT71; Table 1) each containing more than 30% “*Ca.* M. girerdii” by metagenomic 16S rRNA gene microbiome analysis were also sequenced by whole metagenome shotgun sequencing using Titanium FLX pyrosequencing and the protocol described above. Each sample was run on approximately one eighth of a plate, yielded between 185,612 and 222,667 total reads and assembled using Newbler, and aligned to the reference strain. The genomes of “*Ca*. M. girerdii” have been deposited with NCBI under the Bioproject accession numbers PRJNA196996, PRJNA196997, PRJNA196998, and PRJNA196999. The complete genome of “*Ca*. M. girerdii” has been deposited at NCBI under accession number CP007711.

### Genome annotation and metabolic reconstruction

Open reading frames (ORFs) greater than 100 nucleotides were predicted by Glimmer3 [56] and GeneMarkS [57] using translation table 4 and were manually examined. In most cases the start site predicted by Glimmer3 was chosen for genes that had the same predicted stop codon called by both Glimmer3 and GeneMarkS. Translated ORF predictions were searched against the non-redundant (nr) database from NCBI and a custom database of Mollicute proteins downloaded from NCBI using the blastp algorithm, and the gene products were manually annotated. Predicted gene products were compared to conserved domain databases (COGs and Pfam) by RPS-BLAST. Other annotation features were predicted using TMHMM 2.0c [58] for transmembrane domains and SignalP 4.0 for signal peptides. Although mollicutes have a unique membrane composition, SignalP has been previously validated on experimentally verified secreted proteins from mollicutes [59]. The hmmsearch program from HMMER3.0 [60] was used to search predicted proteins for the *Treponema palladium* family of leucine rich repeats (TpLRR) using the Pfam raw hidden markov model for the family (LRR_5; PF13306). Transfer RNA genes were predicted by tRNAscan-SE v 1.3 using the genetic code outlined in translation table 4. The tRNA-Ile, elongator tRNA-Met and initiator tRNA-fMet were distinguished by alignment with previously annotated tRNAs [61]. The CRISPR element containing four 34-nucleotide repeats and three 35-nucleotide spacers was identified in the genome with a consensus direct repeat sequence of 5′-AAGTATTAATATTCCAAGTAGTGTAACTAGTATT using the CRISPR recognition tool (CRT) [62]. Metabolic reconstruction and Gene Ontology classification assignments were performed using ASGARD [63] and the UniRef100 database.

### Phylogenetic analysis

One reference genome was selected for each species in the Mollicutes class for which a completely sequenced genome is available. A total of 57 transitively closed orthologous clusters were retrieved from the RoundUp [64] database (release date: Dec. 23, 2011). “*Ca.* M. girerdii” orthologs were identified using blastp and confirmed using the Reciprocal Smallest Distance (RSD) algorithm. Orthologs were similarly identified for *M. iowae* for which only a draft genome is available. The maximum-likelihood tree was inferred by RAxML 7.2.74 [65] using the gamma-distributed heterogeneity rate categories with 1,000 bootstraps. The tree was rooted using *Lactobacillus gasseri* as the outgroup. Phylogenetic trees based on 16S rDNA gene sequences were similarly constructed. The 16S rRNA gene alignments were manually inspected and the maximum likelihood tree was inferred by RAxML 7.2.74 using the gamma-distributed heterogeneity rate categories with 1,000 bootstraps.

### Attempts to cultivate “*Ca*. M. girerdii”

Frozen vaginal swab samples were incubated on A8 and SP4 agar (Hardy Diagnostics). When these plates did not yield colonies, the frozen samples were cultured on PPLO broth base containing 10% horse or 10% human serum, 10% yeast extract, 1% arginine and 1.5% Bacto agar [66]. The samples were also incubated on supplemented BHI agar [67] containing 10% human blood. All solid media was supplemented with 100 µg ampicillin/mL and plates were incubated at 37°C under anaerobic conditions and in air supplemented with 5% CO_2_. None of these efforts yielded detectable growth.

### Ethics Statement

Consent was obtained from all participants in accordance the study protocol as approved by the institutional review boards for human subjects protection at Virginia Commonwealth University and the Virginia Department of Health. All enrolled subjects were 18 years of age or older and provided written informed consent.

## Supporting Information

Figure S1 **Cluster analysis of mid-vaginal samples with a clinical diagnosis of trichomoniasis.** Relative abundance of microbial taxa in mid-vaginal bacterial communities of 63 women with clinically diagnosed trichomoniasis is shown. The dendrogram was generated using Ward’s method with Manhattan distance. Presence of “*Ca*. M. girerdii” as determined by 16S rDNA profiling is indicated in the top bar. Samples that contained “*Ca*. M. girerdii” at less than 0.1% abundance are indicated as ambiguous (AMB) in light blue.
(PDF)

Figure S2 **Detection of “** ***Ca*** **. M. girerdii” in mid-vaginal samples.** Fluorescence *in situ* hybridization detection of bacteria in mid-vaginal samples from two participants with clinically diagnosed trichomoniasis (subject 1, panels A–O); subject 2, panels P–T) by confocal laser scanning microscopy. The merged photomicrographs are also depicted in Figure 1. Nuclear DNA was detected with 4′6′-diamidine-2-phenylindole, dehydrochloride (DAPI, blue) as shown in panel B, G, L and Q. Most bacteria were detected with fluorescein-labeled broad-range bacteria probe Eub338 (green) as shown in panels C, H, M and R. “*Ca*. M. girerdii” was also stained with a Cy5-labeled probe targeting 16S rDNA (red) as shown in panels D, I, N and S.
(PDF)

Figure S3 ***“Ca.*** **M. girerdii** ***”*** **coexists with both genotypes of** ***T. vaginalis.*** The maximum likelihood tree was constructed using concatentated, aligned partial protein sequences from three single-copy orthologs (CRN, PMS1, Mlh1a). Isolates indicated as type 1 or type 2 were previously typed using microsatellite markers. Strains from 43 clinically diagnosed cases of trichomoniasis from this study are indicated with the prefix “VCU”. The type 1 cluster is shaded blue and contains eight “*Ca*. M. girerdii” positive cases, the type 2 cluster is shaded green and contains ten “*Ca.* M. girerdii*”* positive cases and the ambiguous cluster is shaded gray and contains five “*Ca*. M. girerdii” positive cases. In this analysis, the ambiguous cluster groups with type 2 *T. vaginalis*, but the subgroup contains isolates that were differentially classified as type 1 using microsatellite markers. *T. vaginalis* strains from “*Ca*. M. girerdii” positive cases as determined by 16S rRNA microbiome profiling (0.1% threshold) are indicated with red boxes. Ambiguous cases that were detected at less than 0.1% threshold are denoted with pink boxes. Blue dots denote branches with bootstrap values greater than 50.
(PDF)

Figure S4 **Conserved synteny among “Ca. M. girerdii” strains not shared with related species.** Panel (A) shows dot plot nucleotide-based alignments of the reference “*Ca*. M. girerdii” strain VCU_M1 with contigs from three different “*Ca*. M. girerdii” strains (VCU_PA1, VCU_JB1, VCU_G1). Panel (B) shows dot plot amino-acid based alignments of the reference with three closely related species (*M. iowae*, *M. penetrans* and *U. parvum*). Horizontal grid lines delineate contigs. Nucleotide-based and protein-based alignments were performed using Nucmer and Promer respectively.
(PDF)

Table S1 **Distribution of Clusters of Orthologous Groups (COGS).**
(DOCX)

Table S2 **Putative transporters in “** ***Ca*** **. M. girerdii” strain VCU_M1.**
(DOCX)

Table S3 **List of primers for circularization of “** ***Ca*** **. M. girerdii” genome.**
(DOCX)

Table S4 **Hominis Group orthologs.**
(DOCX)

Table S5 **Pneumoniae and Hemoplasma Group orthologs.**
(DOCX)

Table S6 **Spiroplasma, Phytoplasma and Outgroup orthologs.**
(DOCX)
