# Supplementary figures and images for: An Emerging Mycoplasma Associated with Trichomoniasis, Vaginal Infection and Disease
Source: PLoS One. 2014 Oct 22;9(10):e110943. doi: 10.1371/journal.pone.0110943 (PMC4206474; doi:10.1371/journal.pone.0110943)

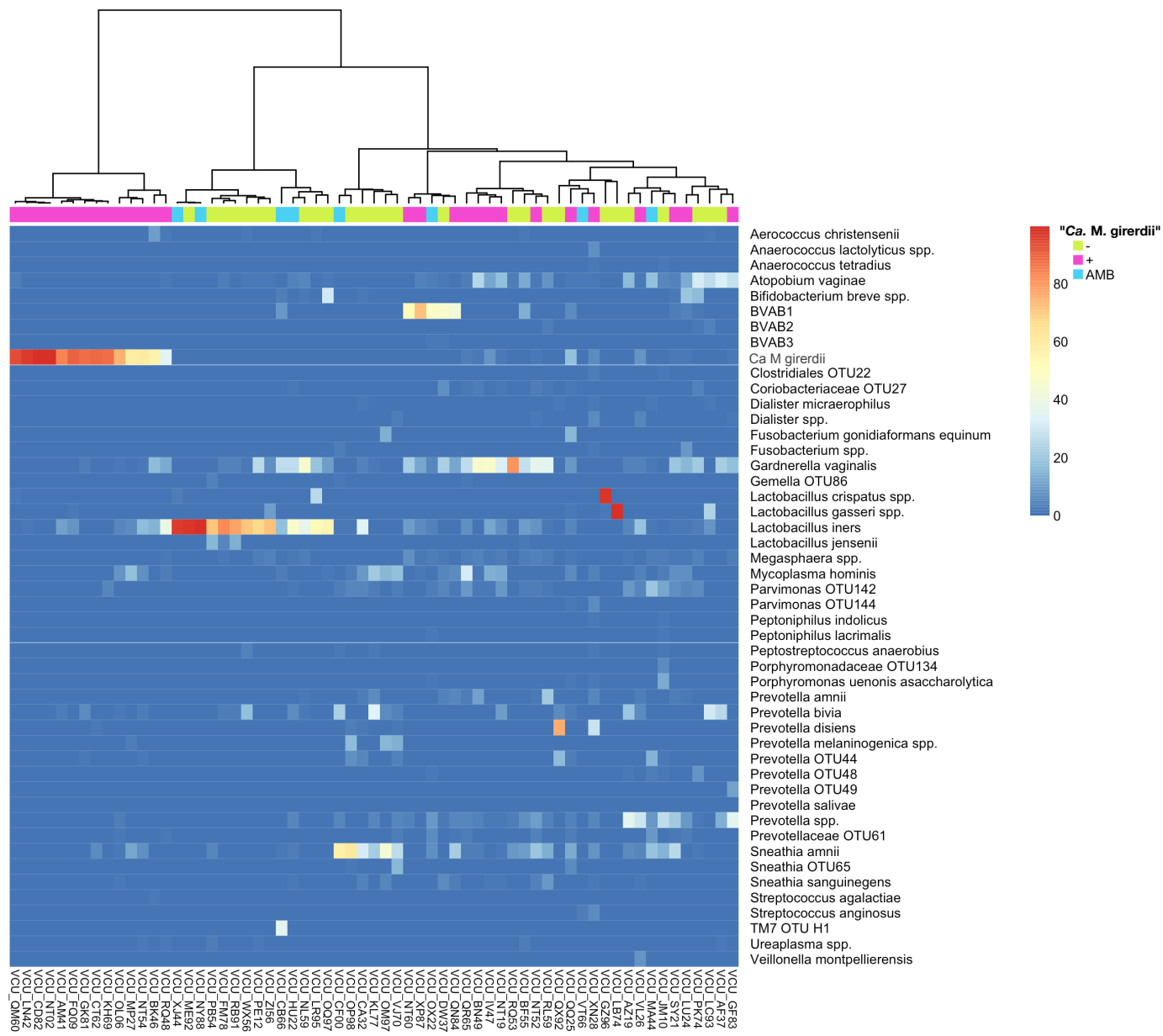

Supplement: Figure S1 — Cluster analysis of mid-vaginal samples with a clinical diagnosis of trichomoniasis. Relative abundance of microbial taxa in mid-vaginal bacterial communities of 63 women with clinically diagnosed trichomoniasis is shown. The dendrogram was generated using Ward’s method with Manhattan distance. Presence of “Ca. M. girerdii” as determined by 16S rDNA profiling is indicated in the top bar. Samples that contained “Ca. M. girerdii” at less than 0.1% abundance are indicated as ambiguous (AMB) in light blue. (PDF) [file pone.0110943.s001.pdf]

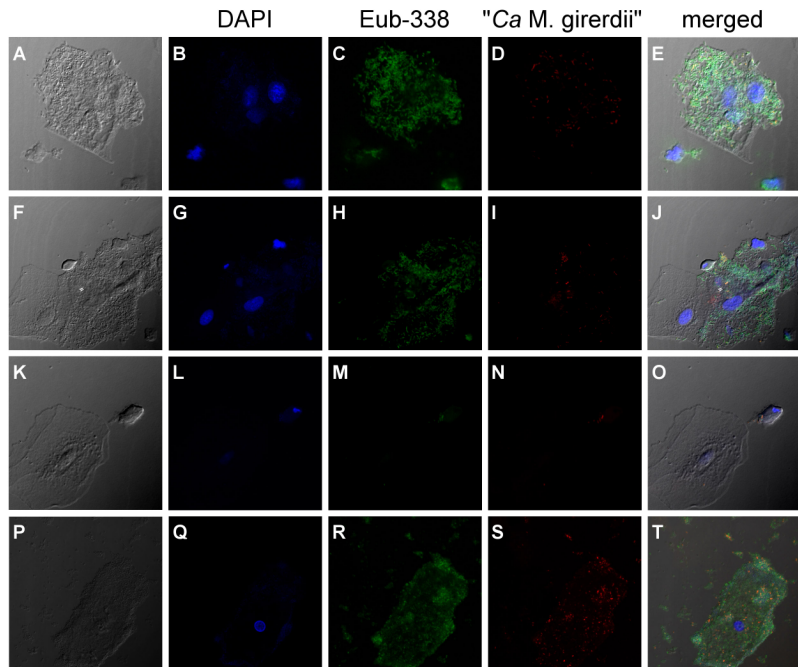

Supplement: Figure S2 — Detection of “ Ca . M. girerdii” in mid-vaginal samples. Fluorescence in situ hybridization detection of bacteria in mid-vaginal samples from two participants with clinically diagnosed trichomoniasis (subject 1, panels A–O); subject 2, panels P–T) by confocal laser scanning microscopy. The merged photomicrographs are also depicted in Figure 1. Nuclear DNA was detected with 4′6′-diamidine-2-phenylindole, dehydrochloride (DAPI, blue) as shown in panel B, G, L and Q. Most bacteria were detected with fluorescein-labeled broad-range bacteria probe Eub338 (green) as shown in panels C, H, M and R. “Ca. M. girerdii” was also stained with a Cy5-labeled probe targeting 16S rDNA (red) as shown in panels D, I, N and S. (PDF) [file pone.0110943.s002.pdf]

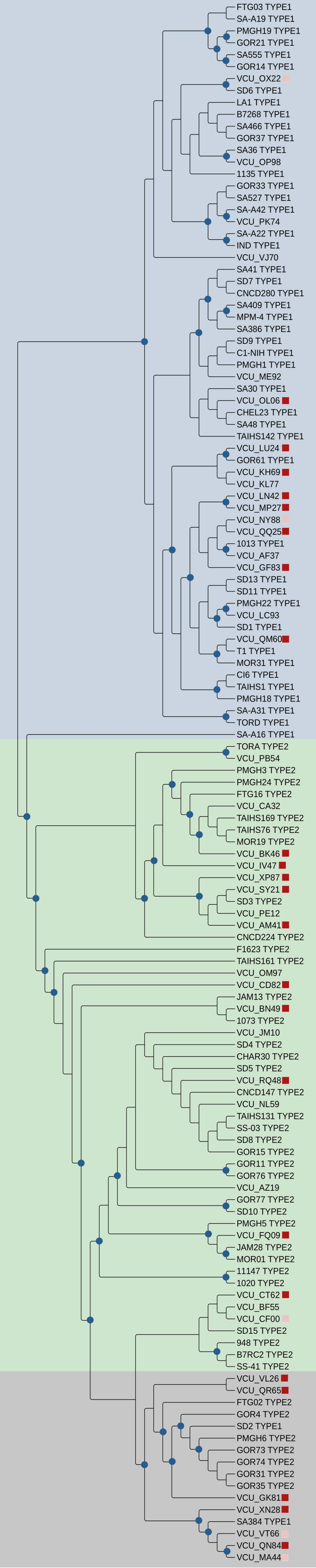

Supplement: Figure S3 — “Ca. M. girerdii ” coexists with both genotypes of T. vaginalis. The maximum likelihood tree was constructed using concatentated, aligned partial protein sequences from three single-copy orthologs (CRN, PMS1, Mlh1a). Isolates indicated as type 1 or type 2 were previously typed using microsatellite markers. Strains from 43 clinically diagnosed cases of trichomoniasis from this study are indicated with the prefix “VCU”. The type 1 cluster is shaded blue and contains eight “Ca. M. girerdii” positive cases, the type 2 cluster is shaded green and contains ten “Ca. M. girerdii” positive cases and the ambiguous cluster is shaded gray and contains five “Ca. M. girerdii” positive cases. In this analysis, the ambiguous cluster groups with type 2 T. vaginalis, but the subgroup contains isolates that were differentially classified as type 1 using microsatellite markers. T. vaginalis strains from “Ca. M. girerdii” positive cases as determined by 16S rRNA microbiome profiling (0.1% threshold) are indicated with red boxes. Ambiguous cases that were detected at less than 0.1% threshold are denoted with pink boxes. Blue dots denote branches with bootstrap values greater than 50. (PDF) [file pone.0110943.s003.pdf]

**A**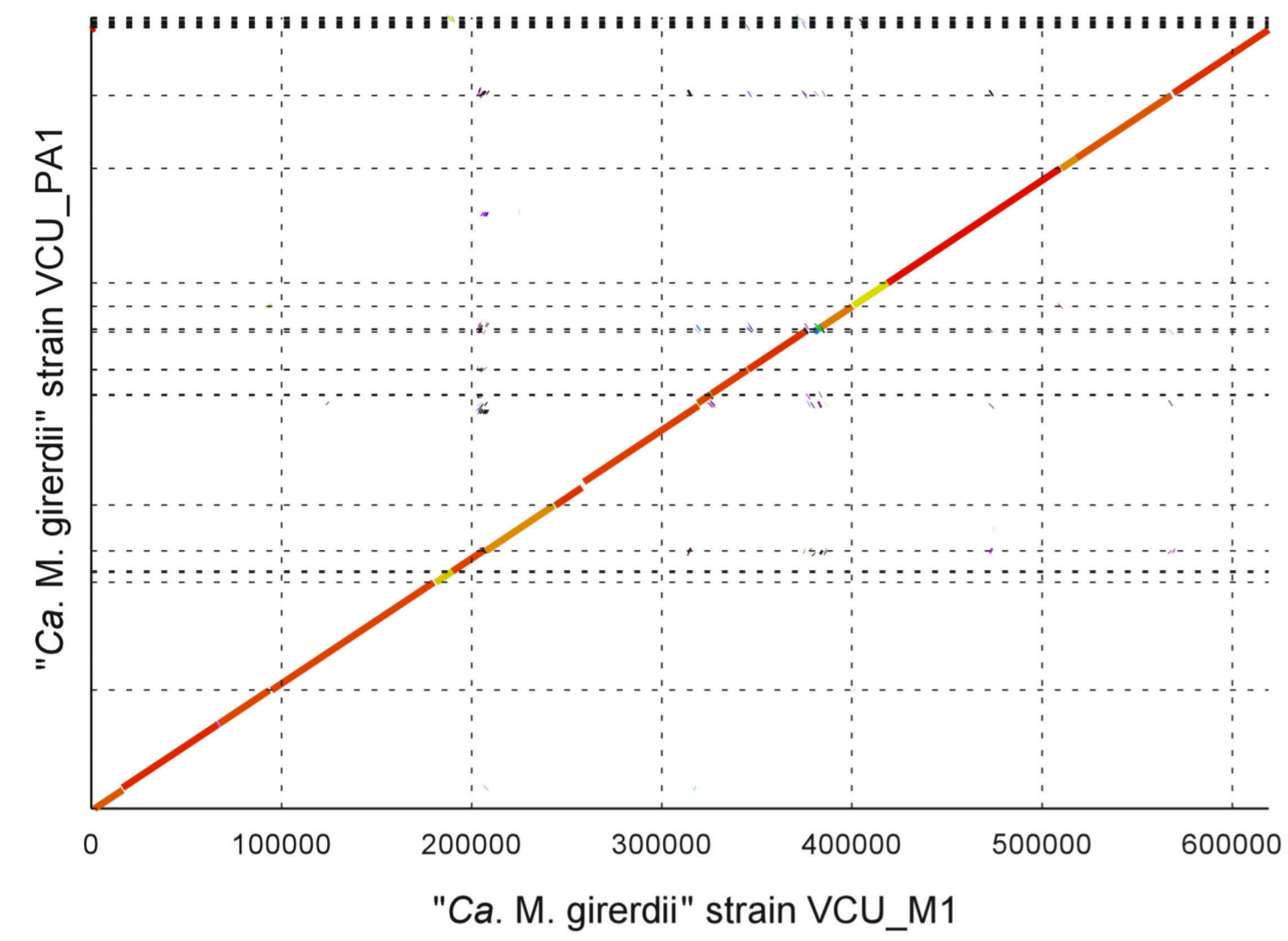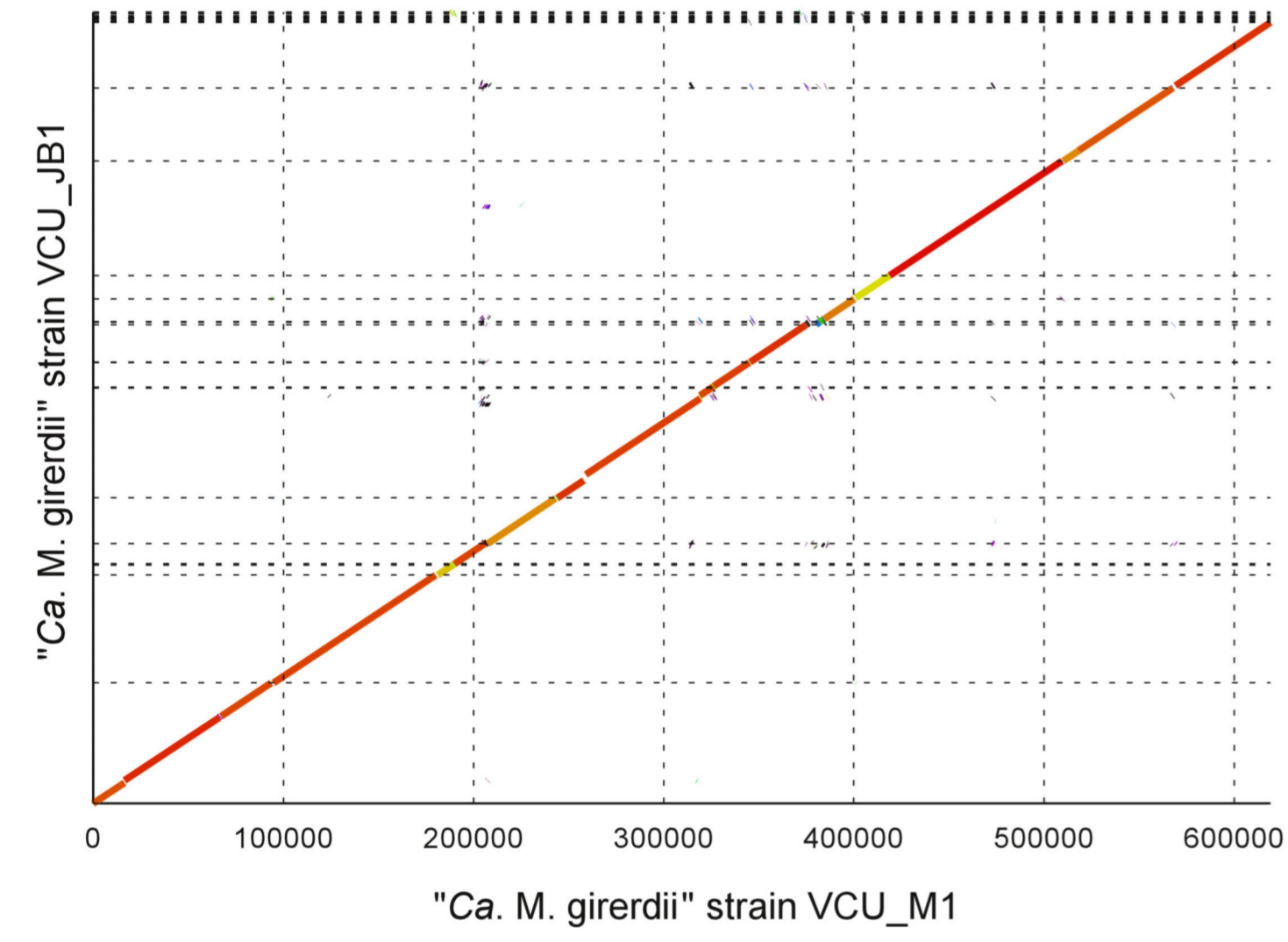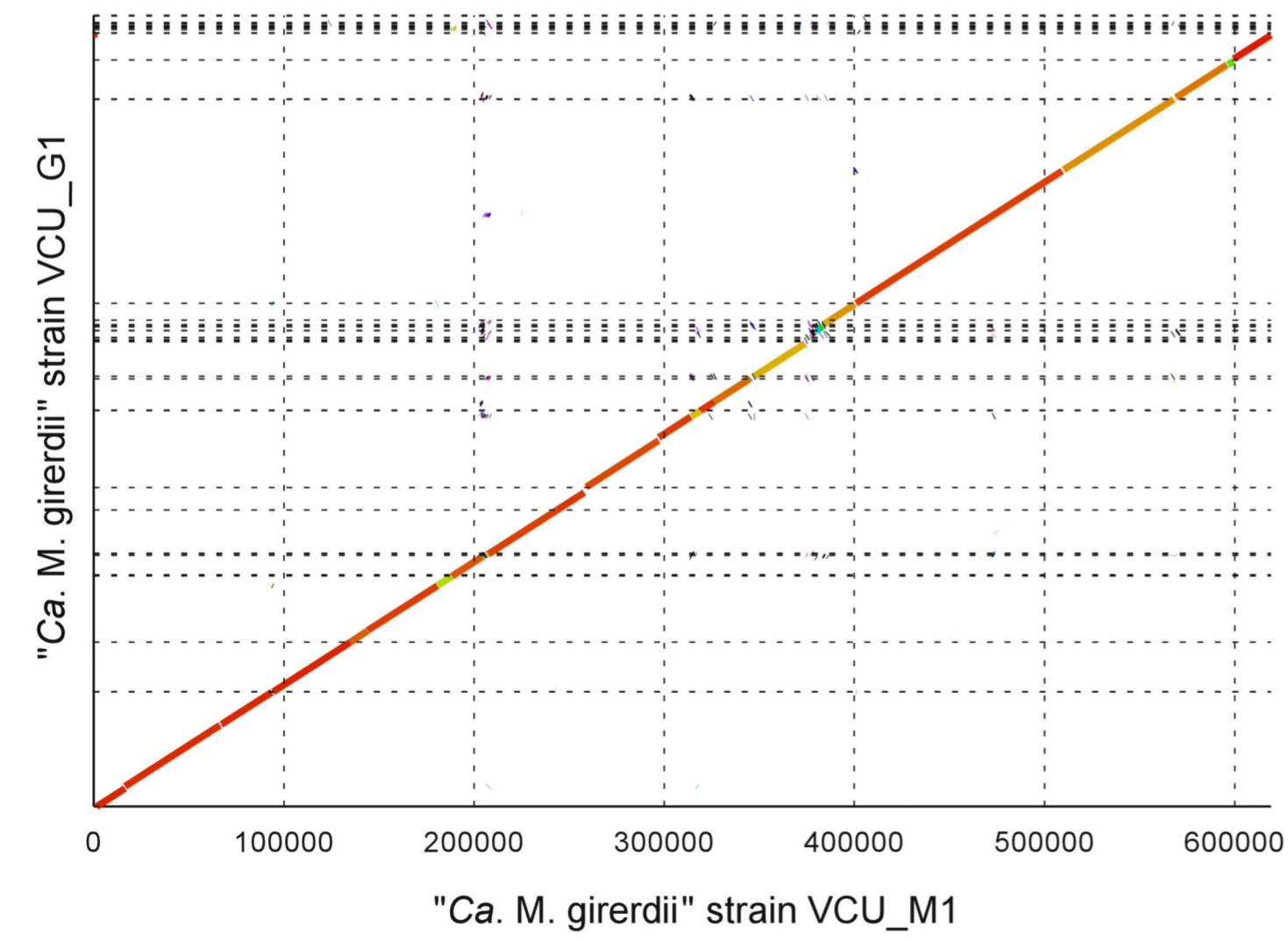**B**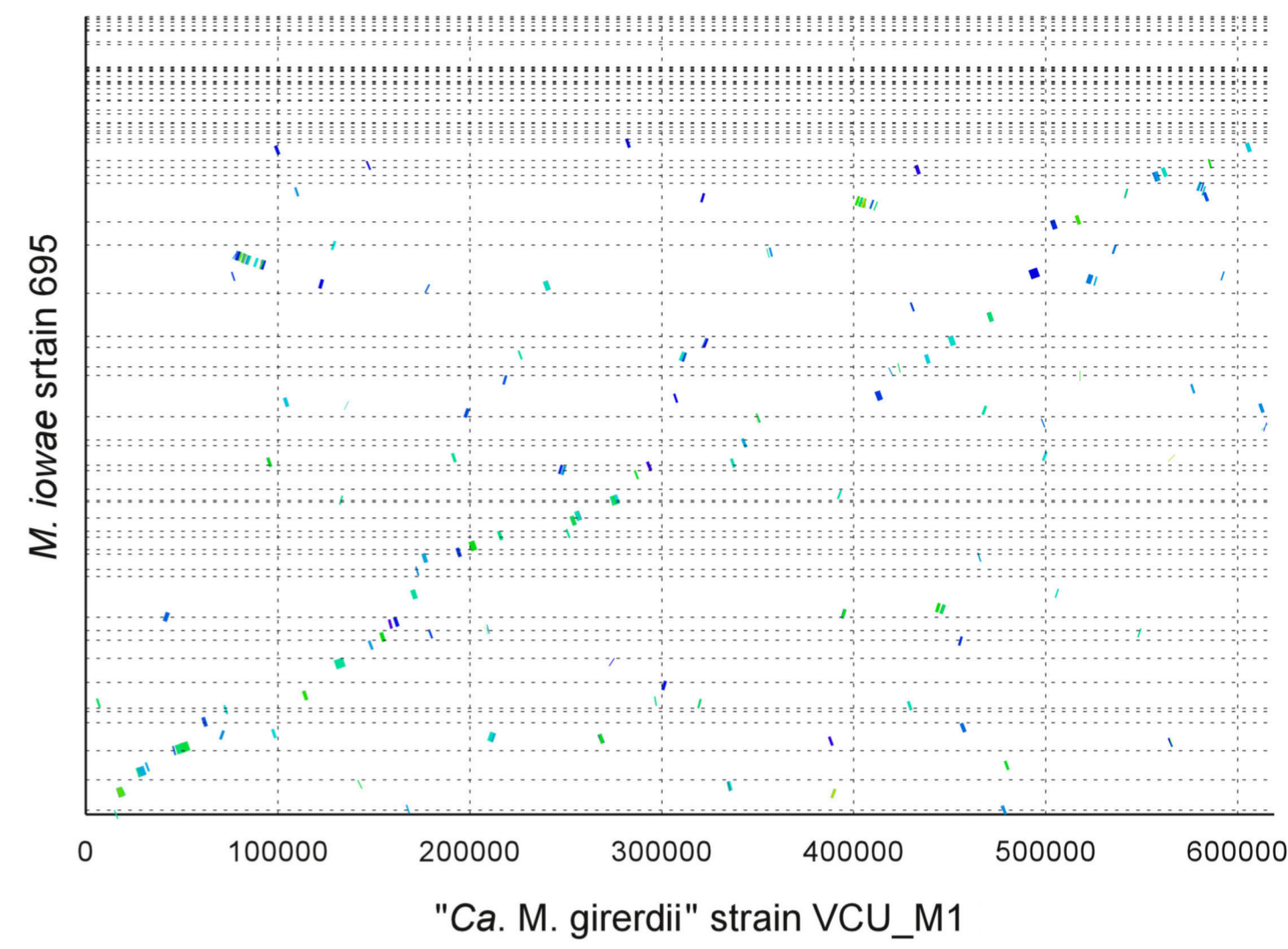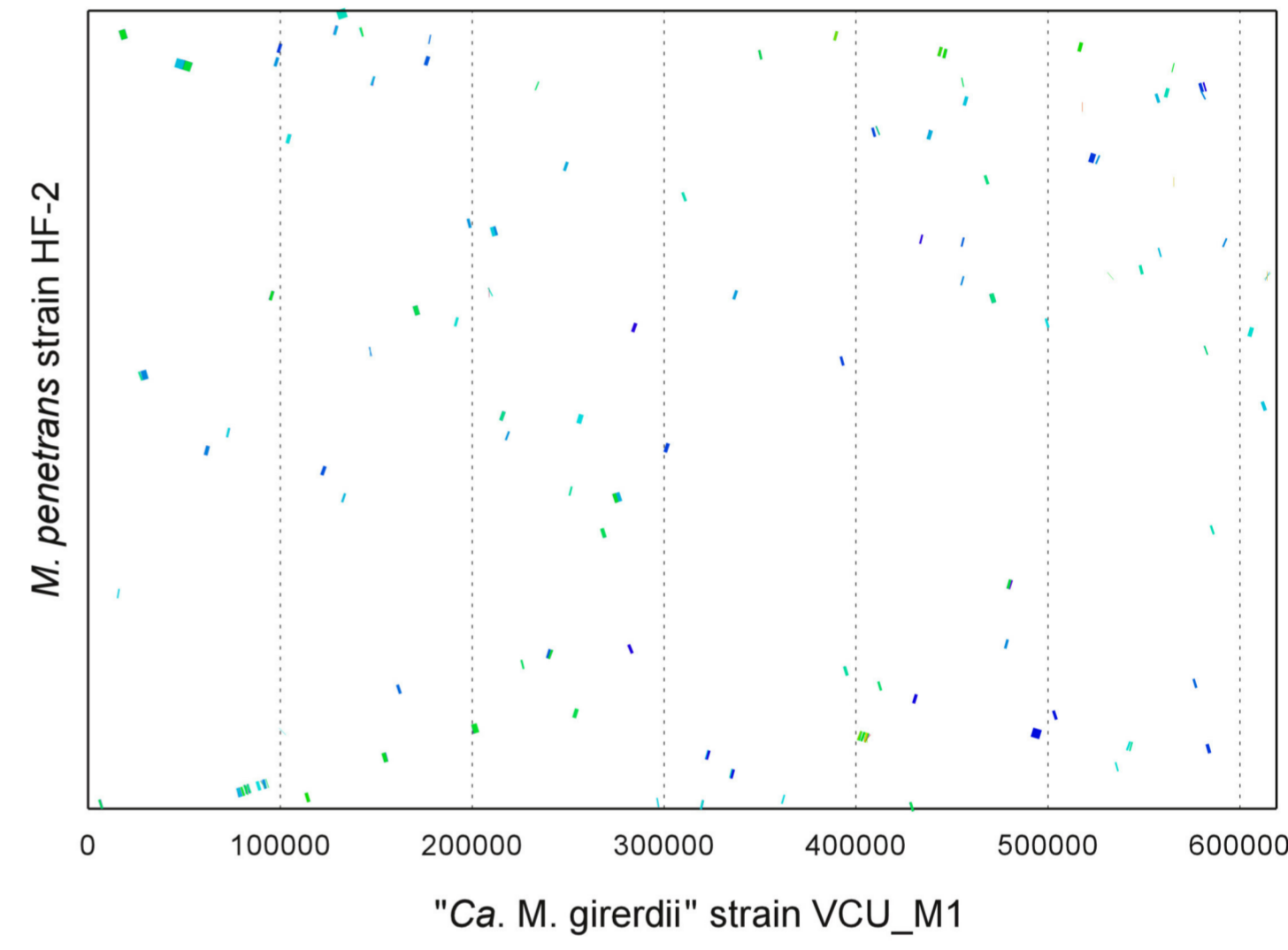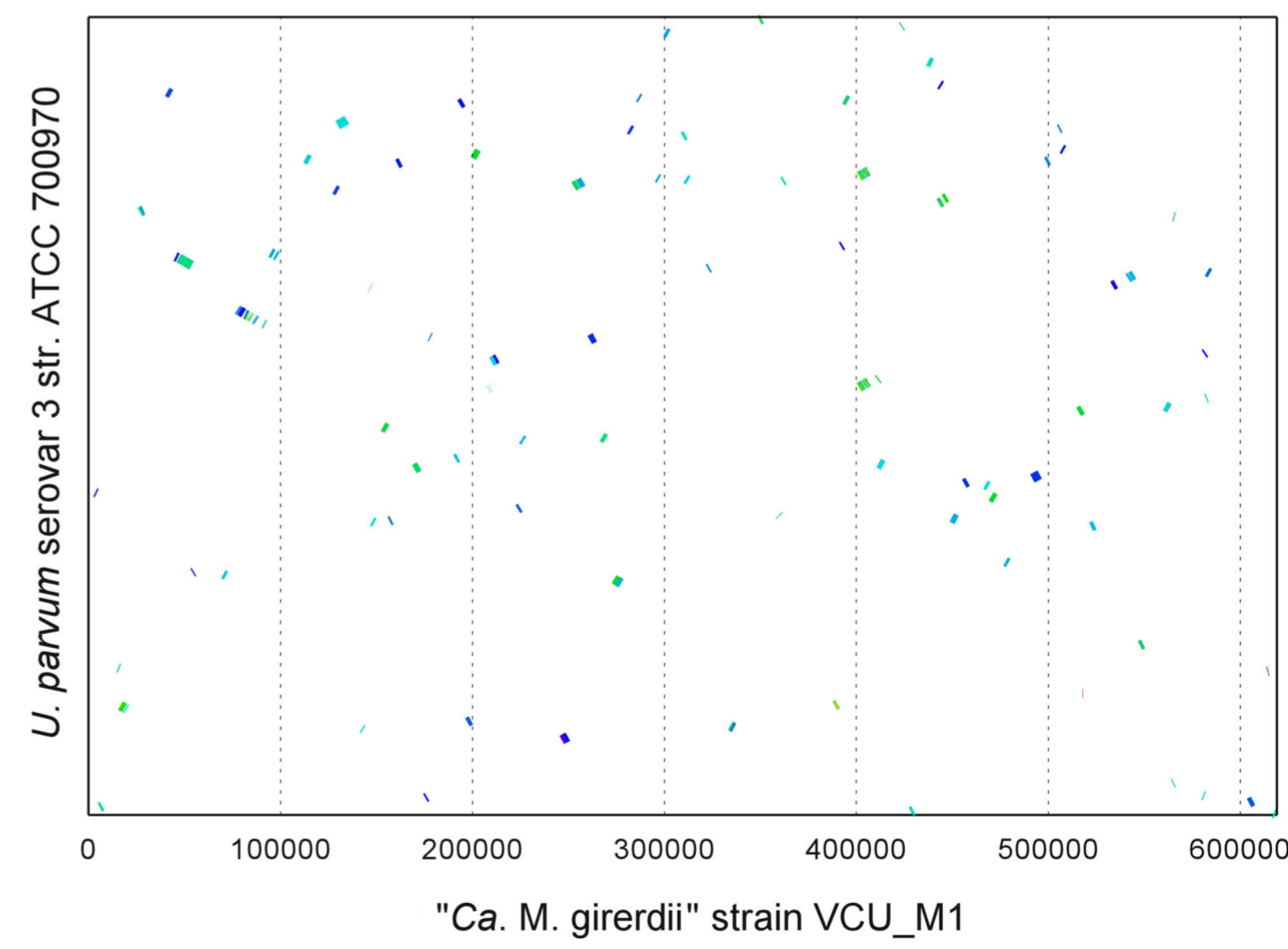

Supplement: Figure S4 — Conserved synteny among “Ca. M. girerdii” strains not shared with related species. Panel (A) shows dot plot nucleotide-based alignments of the reference “Ca. M. girerdii” strain VCU_M1 with contigs from three different “Ca. M. girerdii” strains (VCU_PA1, VCU_JB1, VCU_G1). Panel (B) shows dot plot amino-acid based alignments of the reference with three closely related species (M. iowae, M. penetrans and U. parvum). Horizontal grid lines delineate contigs. Nucleotide-based and protein-based alignments were performed using Nucmer and Promer respectively. (PDF) [file pone.0110943.s004.pdf]
