# Supplementary material for: An Emerging Mycoplasma Associated with Trichomoniasis, Vaginal Infection and Disease
Source: PLoS One. 2014 Oct 22;9(10):e110943. doi: 10.1371/journal.pone.0110943 (PMC4206474; doi:10.1371/journal.pone.0110943)
Supplement: Table S1 — Distribution of Clusters of Orthologous Groups (COGS). (DOCX) [file pone.0110943.s005.docx]

**Table S1. Distribution of Clusters of Orthologous Groups (COGS).**

|  | “*Ca*. Mycoplasma girerdii” | *M. iowae* | *M. penetrans* | *U. urealyticum* | *U. parvum* | *M. hominis* | *M. genitalium* |
| --- | --- | --- | --- | --- | --- | --- | --- |
| [J] Translation, ribosomal structure and biogenesis | 105 | 107 | 106 | 105 | 103 | 106 | 99 |
| [K] Transcription | 16 | 22 | 29 | 19 | 20 | 16 | 16 |
| [L] Replication, recombination and repair | 44 | 58 | 75 | 49 | 50 | 45 | 38 |
| [D] Cell cycle control, cell division, chromosome partitioning | 7 | 16 | 9 | 6 | 7 | 9 | 8 |
| [M] Cell wall/membrane/envelope biogenesis | 8 | 16 | 18 | 11 | 8 | 12 | 14 |
| [N] Cell motility | 0 | 1 | 0 | 0 | 0 | 0 | 0 |
| [O] Posttranslational modification, protein turnover, chaperones | 21 | 25 | 28 | 19 | 19 | 14 | 20 |
| [T] Signal transduction mechanisms | 2 | 8 | 6 | 4 | 3 | 2 | 4 |
| [U] Intracellular trafficking, secretion, and vesicular transport | 7 | 7 | 7 | 9 | 8 | 7 | 8 |
| [V] Defense mechanisms | 19 | 24 | 27 | 14 | 14 | 18 | 9 |
| [C] Energy production and conversion | 24 | 30 | 31 | 17 | 17 | 18 | 20 |
| [E] Amino acid transport and metabolism | 14 | 31 | 29 | 22 | 22 | 23 | 15 |
| [F] Nucleotide transport and metabolism | 27 | 40 | 38 | 23 | 23 | 19 | 21 |
| [G] Carbohydrate transport and metabolism | 29 | 56 | 50 | 15 | 14 | 19 | 25 |
| [H] Coenzyme transport and metabolism | 12 | 22 | 14 | 9 | 9 | 7 | 13 |
| [I] Lipid transport and metabolism | 7 | 17 | 17 | 8 | 8 | 7 | 8 |
| [P] Inorganic ion transport and metabolism | 12 | 32 | 22 | 29 | 27 | 9 | 17 |
| [Q] Secondary metabolites biosynthesis, transport and catabolism | 1 | 3 | 2 | 0 | 0 | 1 | 0 |
| [R] General function prediction only | 37 | 70 | 88 | 46 | 46 | 32 | 40 |
| [S] Function unknown | 23 | 39 | 27 | 24 | 28 | 22 | 15 |
| Not in COGs | 157 | 357 | 414 | 217 | 188 | 137 | 85 |
