# Supplementary material for: An Emerging Mycoplasma Associated with Trichomoniasis, Vaginal Infection and Disease
Source: PLoS One. 2014 Oct 22;9(10):e110943. doi: 10.1371/journal.pone.0110943 (PMC4206474; doi:10.1371/journal.pone.0110943)
Supplement: Table S2 — Putative transporters in “ Ca . M. girerdii” strain VCU_M1. (DOCX) [file pone.0110943.s006.docx]

**Table S2. Putative transporters in “*Ca*. M. girerdii” strain VCU_M1.**

| **Orf** | **Annotation** | **Transporter Class** |
| --- | --- | --- |
| MGM1_1730 | putative permease, major facilitator superfamily | The Major Facilitator Superfamily (MFS) |
| MGM1_1430 | amino acid permease | The Amino Acid-Polyamine-Organocation (APC) Superfamily |
| MGM1_5950 | amino acid permease | The Amino Acid-Polyamine-Organocation (APC) Superfamily |
| MGM1_5930 | amino acid permease | The Amino Acid-Polyamine-Organocation (APC) Superfamily |
| MGM1_0670 | Cation transport protein TrkG | The K^+^ Transporter (Trk) Family |
| MGM1_0680 | Trk system potassium uptake protein TrkA | The K^+^ Transporter (Trk) Family |
| MGM1_3060 | ribose/galactose ABC transporter | The ATP-binding Cassette (ABC) Superfamily |
| MGM1_3070 | ribose/galactose ABC transporter | The ATP-binding Cassette (ABC) Superfamily |
| MGM1_3080 | ribose/galactose ABC transporter ATP-binding protein | The ATP-binding Cassette (ABC) Superfamily |
| MGM1_0340 | ABC transporter ATP-binding protein | The ATP-binding Cassette (ABC) Superfamily |
| MGM1_0360 | ABC transporter ATP-binding protein | The ATP-binding Cassette (ABC) Superfamily |
| MGM1_0370 | ABC-2 type transporter | The ATP-binding Cassette (ABC) Superfamily |
| MGM1_2920 | Multidrug resistance ABC transporter ATP-binding protein | The ATP-binding Cassette (ABC) Superfamily |
| MGM1_2930 | Multidrug resistance ABC transporter ATP-binding protein | The ATP-binding Cassette (ABC) Superfamily |
| MGM1_3670 | ABC-type antimicrobial peptide transport system ATPase | The ATP-binding Cassette (ABC) Superfamily |
| MGM1_3690 | ABC-type antimicrobial peptide transport system permease | The ATP-binding Cassette (ABC) Superfamily |
| MGM1_3400 | ABC-type cobalt transport system ATPase | The ATP-binding Cassette (ABC) Superfamily |
| MGM1_3420 | ABC-type cobalt transport system ATPase | The ATP-binding Cassette (ABC) Superfamily |
| MGM1_3390 | ABC-type cobalt transport system permease component | The ATP-binding Cassette (ABC) Superfamily |
| MGM1_5260 | ABC-type phosphate transport system ATPase PstB | The ATP-binding Cassette (ABC) Superfamily |
| MGM1_5270 | ABC-type phosphate transport system permease PstA | The ATP-binding Cassette (ABC) Superfamily |
| MGM1_5280 | ABC-type phosphate transport system substrate-binding protein PstS | The ATP-binding Cassette (ABC) Superfamily |
| MGM1_5290 | ABC-type bacteriocin/lantibiotic exporter | The ATP-binding Cassette (ABC) Superfamily |
| MGM1_1660 | putative spermidine/putrescine-binding periplasmic transport protein (potD) | The ATP-binding Cassette (ABC) Superfamily |
| MGM1_1670 | spermidine/putrescine transport system permease PotC | The ATP-binding Cassette (ABC) Superfamily |
| MGM1_1680 | spermidine/putrescine transport system permease PotB | The ATP-binding Cassette (ABC) Superfamily |
| MGM1_1690 | spermidine/putrescine transport ATP-binding protein PotA | The ATP-binding Cassette (ABC) Superfamily |
| MGM1_4420 | ABC transporter permease | The ATP-binding Cassette (ABC) Superfamily |
| MGM1_4430 | ABC transporter ATP-binding protein | The ATP-binding Cassette (ABC) Superfamily |
| MGM1_3460 | putative ABC-2 family transporter | The ATP-binding Cassette (ABC) Superfamily |
| MGM1_4460 | calcium ion/cation transport ATPase | The P-type ATPase (P-ATPase) Superfamily |
| MGM1_1240 | magnesium-importing ATPase | The P-type ATPase (P-ATPase) Superfamily |
| MGM1_0530 | phosphotransferase system component EIIA | The PTS Glucose-Glucoside (Glc) Family |
| MGM1_0540 | phosphotransferase system component EIIBC | The PTS Glucose-Glucoside (Glc) Family |
| MGM1_1120 | glucuose-specific phosphotransferase system component EIIABC | The PTS Glucose-Glucoside (Glc) Family |
| MGM1_2520 | glucose-specific phosphotransferase system component EIIABC | The PTS Glucose-Glucoside (Glc) Family |
| MGM1_4770 | lactose-specific phosphotransferase enzyme IIA component | The PTS Lactose-N,N'-Diacetylchitobiose-β-glucoside (Lac) Family |
| MGM1_2260 | PTS system enzyme I | The Phosphotransferase System Enzyme I (EI) Family |
| MGM1_1420 | phosphocarrier HPr protein | The Phosphotransferase System HPr (HPr) Family |
| MGM1_3700 | FeoA domain-containing protein | The Ferrous Iron Uptake (FeoB) Family |
| MGM1_3710 | ferrous iron transport protein B FeoB | The Ferrous Iron Uptake (FeoB) Family |
