# Supplementary material for: An Emerging Mycoplasma Associated with Trichomoniasis, Vaginal Infection and Disease
Source: PLoS One. 2014 Oct 22;9(10):e110943. doi: 10.1371/journal.pone.0110943 (PMC4206474; doi:10.1371/journal.pone.0110943)
Supplement: Table S3 — List of primers for circularization of “ Ca . M. girerdii” genome. (DOCX) [file pone.0110943.s007.docx]

**Table S3. List of primers for circularization of “*Ca*. M. girerdii” genome.**

| Type | Primer Name | Sequence (5’->3’) |
| --- | --- | --- |
| forward primer | M1_Contig2-231-7_F | TGGTTCAATGTGAGAGCAATC |
| reverse primer | M1_Contig2-231-7_R | TGACTTCAAAATCTTCTTCACTGT |
| forward primer | M1_Contig7-461-1_F | TGGCTTAATTTTAGCGAAAGAAGG |
| reverse primer | M1_Contig7-461-1_R | AACATCACCTTTAGTTCCGCAG |
| forward primer | M1_Contig1-3_F | TGCATATCCATCTAATGCAACC |
| reverse primer | M1_Contig1-3_R | AGGGACTGTATTTTATCGCAATGG |
| forward primer | M1_Contig3-231-5_F | TTGTGGAGAAAGAAAGCCAAAAAC |
| reverse primer | M1-Contig3-231-5_R | CCAACAAAAAGATTTCTCCCATTC |
| forward primer | M1-Contig5-4_F | TTGAAGGTTCGATTCCTTTAGTCG |
| reverse primer | M1-Contig5-4_R | GTAGCAGATGAATAAACAGGATATTGC |
| forward primer | M1-Contig4-1409-10_F | AATTCGATCAGCCATTCTATTTGG |
| reverse primer | M1-Contig4-1409-10_R | GATTCTTGGTGGGAACTTACAG |
| forward primer | M1_Contig1409-10_F2 | GCTGTAAGTTCCCACCAAG |
| reverse primer | M1_Contig1409-10_R2 | CAAAGCAATGCATACAAGTGAG |
| forward primer | M1-10-X-6_F | TTTGTGCAATGTATTCGCCCTATG |
| reverse primer | M1-10X-6_R | ATGGAAGCCACTTTCCTTTTGATG |
| forward primer | M1-6-117_F | GTTTAGAGACATCAAGGCCAG |
| reverse primer | M1-6-117_R | ATACTTGTTAATGCACTGCAACTATC |
| forward primer | M1-117-2_F | TTAAAGGTTGCAGTTCCCTAAT |
| reverse primer | M1-117-2_R | AGAGATGCTAATGGTAAATACGTAAGA |
| reverse primer | M1-117-2_R2 | CGTGGTTGTAATTCGTTAGTAAG |
| forward primer* | M1-8-8_F | TTACGCCAAATCTAGTAAAACCATTG |
| reverse primer* | M1-8-8_R | ACTAATTTCCCAATACCCAGTACC |
| sequencing primer | M1_Contig7-461-1_Internal_F | AGAGCTTTTGATAGTTGCAGTG |
| sequencing primer | M1_Contig7-461-1_Internal_R | TTTGATTAATTGCTCCTCATTCC |
| sequencing primer | M1-10-X-6_Internal_F1 | CAATATACAGGAACATTTACTCGATAC |
| sequencing primer | M1-10-X-6_Internal_R1 | GGAACTCAATCATAAGTTTGATTTC |
| sequencing primer | M1-10-X-6_Internal_F2 | TGTAGTGGAATAACTCATATCGG |
| sequencing primer | M1-10-X-6_Internal_R2 | GTTAATGAAGTACAACTTTCAAAAGC |
| sequencing primer | M1-10-X-6_Internal_F3 | AGAAGTGTTAGTTCTTCTGTTGC |
| sequencing primer | M1-10-X-6_Internal_R3 | CATCATAAGTACATAATCCTCCAAC |
| sequencing primer | M1-10-X-6_Internal_F4 | TTATCAGTATTGGTCATAATGCG |
| sequencing primer | M1-10-X-6_Internal_R4 | TAAGATAATTGAACCAAAGATTAAACC |
| sequencing primer | M1-6-117_Internal_F | CAAATTAACTAGTATTGCTATAGGGAC |
| sequencing primer | M1_Contig2-231-7_Internal_F | TGTTTTGATTACAAAATCAGAAACAC |
| sequencing primer | M1_Contig1409-10_F2 | GCTGTAAGTTCCCACCAAG |
| sequencing primer | M1_Contig1409-10_R2 | CAAAGCAATGCATACAAGTGAG |

Note: Amplification primers were also used for sequencing.

*Primers used to circularize the plasmid.
