# Supplementary material for: An Emerging Mycoplasma Associated with Trichomoniasis, Vaginal Infection and Disease
Source: PLoS One. 2014 Oct 22;9(10):e110943. doi: 10.1371/journal.pone.0110943 (PMC4206474; doi:10.1371/journal.pone.0110943)
Supplement: Table S4 — Hominis Group orthologs. (DOCX) [file pone.0110943.s008.docx]

**Table S4. Hominis Group orthologs.**

| **Gene** | ***Mycoplasma arthritidis* 158L3-1** | ***Mycoplasma synoviae* 53** | ***Mycoplasma mobil*e 163K** | ***Mycoplasma pulmonis* UAB CTIP** | ***Mycoplasma bovis* PG45** | ***Mycoplasma hyopneumoniae* 232** | ***Mycoplasma hominis* ATCC 23114** | ***Mycoplasma agalactiae* PG2** | ***Mycoplasma crocodyli* MP145** | ***Mycoplasma conjunctivae* HRC/581** | ***Mycoplasma fermentans* JER** | ***Mycoplasma hyorhinis* HUB-1** |
| --- | --- | --- | --- | --- | --- | --- | --- | --- | --- | --- | --- | --- |
| engA | B3PME2 | Q4A6Q9 | Q6KHG1 | Q98RC1 | E4Q0Y9 | Q601X6 | D1J7V5 | A5IZG5 | D5E673 | C5J631 | E1PRX1 | E0TK96 |
| dnaJ | B3PMD7 | Q4A5H1 | Q6KHF9 | Q98PI9 | E4Q0Z2 | Q601X8 | D1J7U9 | A5IZG9 | D5E4M0 | C5J755 | E1PRU2 | E0TK61 |
| cbiO2 | B3PMJ3 | Q4A5A5 | Q6KHL1 | Q98QH5 | E4PZL0 | Q601T5 | D1J7Y4 | A5IYV7 | D5E692 | C5J6Q9 | E1PT60 | E0TKD4 |
| tuf | B3PMU1 | Q4A597 | Q6KI66 | Q98QG1 | E4PZX2 | Q600B6 | P22679 | A5IYA9 | D5E4N2 | C5J6Y9 | E1PS26 | E0TL73 |
| gyrA | B3PN30 | Q4A691 | Q6KHF0 | Q98R63 | E4PZG3 | Q600B1 | D1J8L4 | A5IZ04 | D5E4S4 | C5J5K8 | E1PSD3 | E0TKK7 |
| hrcA | B3PN01 | Q4A656 | Q6KIH9 | Q98R68 | E4PZG7 | Q602E0 | D1J8J7 | A5IZ00 | D5E600 | C5J5V9 | E1PSD7 | E0TKB4 |
| infB | B3PNC4 | Q4A578 | Q6KID8 | Q98R05 | E4Q0V2 | Q5ZZV6 | D1J8W4 | A5IZD8 | D5E4K7 | C5J6X9 | E1PRR0 | E0TKS9 |
| adk | B3PMM7 | Q4A5I4 | Q6KI35 | Q98Q02 | E4PZK3 | Q601J4 | D1J866 | A5IYW4 | D5E4X8 | C5J5V1 | E1PT68 | E0TK22 |
| gmk | B3PN74 | Q4A6S7 | Q6KH87 | Q98PN5 | E4Q0F8 | Q601H3 | D1J8S4 | A5IXZ9 | D5E5A8 | C5J761 | E1PR67 | E0TKE0 |
| mnmA | B3PM68 | Q4A634 | Q6KHK4 | Q98Q11 | E4Q064 | Q600M2 | D1J7R7 | A5IYK6 | D5E527 | C5J5Q2 | E1PSL7 | E0TK62 |
| nusA | B3PNC2 | Q4A579 | Q6KIE0 | Q98R07 | E4Q0V4 | Q5ZZV4 | D1J8W2 | A5IZE0 | D5E4K5 | C5J6Y1 | E1PRQ9 | E0TKT1 |
| pth | B3PNH1 | Q4A5E8 | Q6KHA3 | Q98PE2 | E4Q114 | Q601M5 | D1J8K9 | A5IZI8 | D5E6H9 | C5J6A5 | E1PRJ1 | E0TKX4 |
| rplK | B3PM28 | Q4A5E0 | Q6KIF0 | Q98RJ9 | E4PZ32 | Q600J6 | D1J7M4 | A5IXL9 | D5E619 | C5J666 | E1PRI3 | E0TLF5 |
| rplM | B3PMK1 | Q4A6X4 | Q6KI61 | Q98Q73 | E4PZS5 | Q5ZZN5 | D1J811 | A5IYP4 | D5E5L4 | C5J5I6 | E1PRY5 | E0TKL7 |
| rplN | B3PMN7 | Q4A5D1 | Q6KI45 | Q98PZ2 | E4PZJ1 | Q601K4 | D1J876 | A5IYX6 | D5E4W6 | C5J5U2 | E1PSZ3 | E0TK32 |
| rplO | B3PMM9 | Q4A5D9 | Q6KI37 | Q98Q00 | E4PZJ9 | Q601J6 | D1J868 | A5IYW8 | D5E4X4 | C5J5U9 | E1PSY5 | E0TK24 |
| rplP | B3PMP0 | Q4A5C8 | Q6KI48 | Q98PY9 | E4PZI8 | Q601K7 | D1J879 | A5IYX9 | D5E4W3 | C5J5T9 | E1PSZ6 | E0TK35 |
| rplQ | B3PMM0 | Q4A5J1 | Q6KI28 | Q98Q09 | E4PZK9 | Q601I8 | Q4A548 | A5IYV8 | D5E4Y5 | C5J5V7 | E1PT61 | E0TK17 |
| rplA | B3PM27 | Q4A5E1 | Q6KIF1 | Q98RJ8 | E4PZ33 | Q600J7 | D1J7M5 | A5IXM0 | D5E618 | C5J665 | E1PRI4 | E0TLF6 |
| rplT | B3PM63 | Q4A5F6 | Q6KH18 | Q98QV0 | E4PZQ3 | Q601E4 | D1J7R6 | A5IYR6 | D5E526 | C5J7C1 | E1PSE2 | E0TKV9 |
| rplV | B3PMP2 | Q4A5C6 | Q6KI50 | Q98PY6 | E4PZI6 | Q601K9 | Q8GM55 | A5IYY1 | D5E4W1 | C5J5T7 | E1PSZ8 | E0TK37 |
| rpmA | B3PMF8 | Q4A5L3 | Q6KHY5 | Q98QN2 | E4PZH2 | Q601F3 | D1J852 | A5IYZ5 | D5E613 | C5J5Y7 | E1PT53 | E0TKJ3 |
| rplB | B3PMP4 | Q4A5C4 | Q6KI52 | Q98PY4 | E4PZI4 | Q601L2 | Q8GM57 | A5IYY3 | D5E4V9 | C5J5T5 | E1PT00 | E0TK39 |
| rplC | B3PMP8 | Q4A5C1 | Q6KI55 | Q98PY0 | E4PZI1 | Q601L5 | D1J886 | A5IYY6 | D5E4V6 | C5J5T2 | E1PT03 | E0TK42 |
| rplD2 | B3PMP6 | Q4A5C2 | Q6KI54 | Q98PY2 | E4PZI2 | Q601L4 | D1J885 | A5IYY5 | D5E4V7 | C5J5T3 | E1PT02 | E0TK41 |
| rplE | B3PMN5 | Q4A5D3 | Q6KI43 | Q98PZ4 | E4PZJ3 | Q601K2 | D1J874 | A5IYX4 | D5E4W8 | C5J5U4 | E1PSZ1 | E0TK30 |
| rplF | B3PMN2 | Q4A5D6 | Q6KI40 | Q98PZ7 | E4PZJ6 | Q601J9 | D1J871 | A5IYX1 | D5E4X1 | C5J5U6 | E1PSY8 | E0TK27 |
| ykqC | B3PM13 | Q4A5M1 | Q6KH27 | Q98PL8 | E4Q0X7 | Q601B5 | D1J8C6 | A5IZF3 | D5E620 | C5J795 | E1PRI1 | E0TKF8 |
| rpoA | B3PMM1 | Q4A5J0 | Q6KI29 | Q98Q08 | E4PZK8 | Q601I9 | D1J860 | A5IYV9 | D5E4Y4 | C5J5V6 | E1PT62 | E0TK18 |
| rpsJ | B3PMP9 | Q4A5C0 | Q6KI56 | Q98PX9 | E4PZI0 | O50185 | D1J887 | A5IYY7 | D5E4V5 | C5J5T1 | E1PT04 | E0TK43 |
| rpsL | B3PMF1 | Q4A701 | Q6KHS3 | Q98QD6 | E4Q0L2 | Q601W6 | D1J7T2 | A5IZ35 | D5E6A1 | C5J607 | E1PT80 | E0TKU0 |
| rpsM | B3PMM3 | Q4A5I8 | Q6KI31 | Q98Q06 | E4PZK6 | Q601J1 | D1J862 | A5IYW1 | D5E4Y2 | C5J5V4 | E1PT64 | E0TK19 |
| rpsR | B3PN58 | Q4A5M9 | Q6KI65 | Q98PW0 | E4Q090 | Q600Z8 | D1J7Q1 | A5IY67 | D5E5N2 | C5J6P8 | E1PS86 | E0TKW5 |
| rpsS | B3PMP3 | Q4A5C5 | Q6KI51 | Q98PY5 | E4PZI5 | Q601L1 | Q8GM56 | A5IYY2 | D5E4W0 | C5J5T6 | E1PSZ9 | E0TK38 |
| rpsB | B3PLW6 | Q4A5Z6 | Q6KIB4 | Q98Q36 | E4Q0B0 | Q601Z3 | D1J8T8 | A5IY48 | D5E5E7 | C5J5S1 | E1PSP1 | E0TK53 |
| rpsC | B3PMP1 | Q4A5C7 | Q6KI49 | Q98PY8 | E4PZI7 | Q601K8 | D1J880 | A5IYY0 | D5E4W2 | C5J5T8 | E1PSZ7 | E0TK36 |
| rpsD | B3PN46 | Q4A5T4 | Q6KIM1 | Q98PK6 | E4PZG5 | Q5ZZW4 | D1J7L7 | A5IZ02 | D5E5Z0 | C5J651 | E1PSD5 | E0TKB1 |
| rpsE | B3PMN0 | Q4A5D8 | Q6KI38 | Q98PZ9 | E4PZJ8 | Q601J7 | D1J869 | A5IYW9 | D5E4X3 | C5J5U8 | E1PSY6 | E0TK25 |
| rpsG | B3PMF0 | Q4A702 | Q6KHS4 | Q98QD7 | E4Q0L1 | Q601W7 | D1J7T3 | A5IZ34 | D5E6A0 | C5J608 | E1PT81 | E0TKU1 |
| rpsH | B3PMN3 | Q4A5D5 | Q6KI41 | Q98PZ6 | E4PZJ5 | Q601K0 | D1J872 | A5IYX2 | D5E4X0 | C5J5U5 | E1PSY9 | E0TK28 |
| rpsI | B3PMK0 | Q4A6X3 | Q6KI60 | Q98Q72 | E4PZS4 | Q5ZZN6 | D1J810 | A5IYP5 | D5E5L3 | C5J5I7 | E1PRY6 | E0TKL8 |
| rsmA | B3PLS2 | Q4A645 | Q6KH80 | Q98RJ3 | E4PZ03 | Q5ZZN4 | D1J7E5 | A5IXI9 | D5E4K0 | C5J5I5 | E1PR53 | E0TKL3 |
| secA | B3PM32 | Q4A6S2 | Q6KIK4 | Q98RA6 | E4Q096 | Q601A7 | D1J7M9 | A5IY62 | D5E5H9 | C5J6Z8 | E1PS68 | E0TL80 |
| secY | B3PMM8 | Q4A5I3 | Q6KI36 | Q98Q01 | E4PZK2 | Q601J5 | D1J867 | A5IYW5 | D5E4X7 | C5J5V0 | E1PT69 | E0TK23 |
| alaS | B3PM69 | Q4A633 | Q6KHK5 | Q98Q12 | E4Q063 | Q601M2 | D1J7R8 | A5IYK5 | D5E528 | C5J6A3 | E1PSL6 | E0TKX7 |
| pheS | B3PM48 | Q4A6A0 | Q6KHX3 | Q98QL3 | E4PZ18 | Q601U5 | D1J7Q4 | A5IXK6 | D5E5B8 | C5J6V7 | E1PRT7 | E0TKY3 |
| hisS | B3PMK6 | Q4A5X2 | Q6KI17 | Q98QM8 | E4Q086 | Q601R3 | D1J7X4 | A5IYN9 | D5E5H2 | C5J714 | E1PT24 | E0TKN7 |
| ileS | B3PMY2 | Q4A5K5 | Q6KHN7 | Q98PQ2 | E4Q0E3 | Q602B7 | D1J893 | A5IY09 | D5E509 | C5J6X1 | E1PTB3 | E0TKM5 |
| leuS | B3PM43 | Q4A697 | Q6KHA5 | Q98RB6 | E4Q013 | Q5ZZP0 | D1J894 | A5IYF3 | D5E537 | C5J5X0 | E1PSI2 | E0TL43 |
| metS | B3PM80 | Q4A6A5 | Q6KHX5 | Q50319 | E4Q0G3 | Q600P5 | D1J7S4 | A5IXZ5 | D5E4Z1 | C5J6B9 | E1PR62 | E0TLL5 |
| asnS | B3PNG8 | Q4A5Y7 | Q6KHD7 | Q98PF2 | E4PZV3 | Q600N9 | D1J8N7 | A5IY91 | D5E544 | C5J667 | E1PSQ0 | E0TLF4 |
| serS | B3PNG9 | Q4A5H3 | Q6KIJ8 | Q98RH5 | E4PZ08 | Q601S3 | D1J8K7 | A5IXJ4 | D5E6G3 | C5J6I8 | E1PRC9 | E0TKP4 |
| oppF-valS | B3PLT3 | Q4A6B0 | Q6KID2 | Q98QV4 | E4PZ86 | Q5ZZL4 | D1J7I5 | A5IXS6 | D5E5X1 | C5J7E8 | E1PS00 | E0TJW4 |
| trpS | B3PN66 | Q4A6N3 | Q6KHE3 | Q98PH7 | E4PZZ4 | Q5ZZU8 | D1J7P3 | A5IYD2 | D5E5H5 | C5J799 | E1PSJ6 | E0TLB2 |
| tyrS | B3PMG0 | Q4A5E2 | Q6KH97 | Q98Q81 | E4Q0Z5 | Q601Y5 | D1J850 | A5IZH1 | D5E4N8 | C5J5R2 | E1PRK4 | E0TKK9 |
| ychF | B3PM42 | Q4A5A1 | Q6KH43 | Q98R45 | E4Q033 | Q600Z9 | D1J7J3 | A5IYH1 | D5E4T2 | C5J6P9 | E1PSX7 | E0TKW4 |
| hit | B3PM11 | Q4A6M9 | Q6KIK1 | Q98RK0 | E4Q0H1 | Q600T9 | D1J8D6 | A5IXY8 | D5E6E9 | C5J726 | E1PR46 | E0TLD4 |
