# Supplementary material for: An Emerging Mycoplasma Associated with Trichomoniasis, Vaginal Infection and Disease
Source: PLoS One. 2014 Oct 22;9(10):e110943. doi: 10.1371/journal.pone.0110943 (PMC4206474; doi:10.1371/journal.pone.0110943)
Supplement: Table S5 — Pneumoniae and Hemoplasma Group orthologs. (DOCX) [file pone.0110943.s009.docx]

**Table S5. Pneumoniae and Hemoplasma Group orthologs.**

| **Gene** | **"*Ca.* Mycoplama girerdii"** | ***Mycoplasma genitalium* G37** | ***Mycoplasma penetrans* HF-2** | ***Mycoplasma pneumoniae* M129** | ***Ureaplasma parvum* serovar 3 str. ATCC 700970** | ***Ureaplasma urealyticum* serovar 10 str. ATCC 33699** | ***Mycoplasma suis* KI3806** | ***Mycoplasma gallisepticum* str. R(low)** | ***Mycoplasma haemofelis* str. Langford 1** | ***Mycoplasma iowae* 695** |
| --- | --- | --- | --- | --- | --- | --- | --- | --- | --- | --- |
| engA | MGM1_4540 | P47571 | Q8EWH6 | P75309 | Q9PQA7 | B5ZBM9 | F0V1J2 | Q7NBV2 | E8ZK28 | G4EM19 |
| dnaJ | MGM1_3650 | P47265 | Q8EUM4 | P78004 | Q9PQ82 | B5ZBQ4 | F0V1H4 | D3DEH5 | E8ZK85 | G4EM76 |
| cbiO2 | MGM1_3420 | P47425 | Q8EUF1 | Q50294 | Q9PPV1 | B5ZC47 | F0V3L2 | Q7NAQ6 | E8ZKE6 | G4END2 |
| tuf | MGM1_3860 | P13927 | Q8EX18 | P23568 | P50068 | B5ZC31 | F0V3B0 | P18906 | E8ZK37 | G4ENC1 |
| gyrA | MGM1_1380 | P47250 | Q8EX46 | P22446 | Q9PR63 | B5ZAR1 | F0V2P7 | P47719 | E8ZGH3 | G4ELS7 |
| hrcA | MGM1_1710 | P47447 | Q8EWX9 | P75351 | Q9PQ68 | B5ZBR8 | F0V2M7 | Q7NBC5 | E8ZGI1 | G4EN94 |
| infB | MGM1_5400 | P47388 | Q8EWU0 | P75590 | Q9PQH1 | B5ZBC9 | F0V3H6 | Q7NBZ4 | E8ZJW4 | G4ELK3 |
| adk | MGM1_0910 | P47417 | Q8EUD3 | Q50299 | Q9PQP0 | B5ZB60 | F0V3Q0 | O52352 | E8ZJX9 | G4ELG0 |
| gmk | MGM1_0660 | P47353 | Q8EVJ9 | P75526 | Q9PQS9 | B5ZB21 | F0V3I8 | Q9KX62 | E8ZKR4 | G4ELX2 |
| mnmA | MGM1_2360 | P47537 | Q8CXQ3 | P75365 | Q9PQ88 | B5ZBP8 | F0V3C5 | Q7NBZ0 | E8ZKC5 | G4EM03 |
| nusA | MGM1_5390 | P47387 | Q8EWU2 | P75591 | Q9PQH3 | B5ZBC7 | F0V3H5 | Q7NBZ6 | E8ZJW5 | G4ELK0 |
| pth | MGM1_1140 | P47329 | Q8EWQ8 | P78034 | Q9PR67 | B5ZAQ7 | F0V3E3 | Q8RLD7 | E8ZKB5 | G4ELE5 |
| rplK | MGM1_1470 | P47327 | Q8EX25 | P75550 | Q9PPU9 | B5ZC49 | F0V3B4 | Q8RLD9 | E8ZKT2 | G4END4 |
| rplM | MGM1_5440 | P47657 | Q8EWW9 | P75178 | Q9PPR2 | B5ZC91 | F0V1N8 | Q7NBH5 | E8ZIX4 | G4ELN9 |
| rplN | MGM1_0810 | P47407 | Q8EUC3 | Q50308 | Q9PQQ0 | B5ZB50 | F0V3P0 | O52342 | E8ZJY9 | G4ELH0 |
| rplO | MGM1_0890 | P47415 | Q8EUD1 | Q50300 | Q9PQP2 | B5ZB58 | F0V3P8 | O52350 | E8ZJY1 | G4ELG2 |
| rplP | MGM1_0780 | P47404 | Q8EUB9 | P41204 | Q9PQQ3 | B5ZB47 | F0V3N7 | O52339 | E8ZJZ2 | G4ELH3 |
| rplQ | MGM1_1000 | P47424 | Q8EUD8 | Q59547 | Q9PQN3 | B5ZB67 | F0V1G0 | Q7NAL2 | E8ZJX2 | G4ELF3 |
| rplA | MGM1_1460 | P47328 | Q8EX24 | P78035 | Q9PPV0 | B5ZC48 | F0V3B5 | Q8RLD8 | E8ZKT3 | G4END3 |
| rplT | MGM1_1080 | P47440 | Q8EUK7 | P78023 | Q9PQR2 | B5ZB38 | F0V291 | Q7NBC0 | E8ZKE0 | G4EMT3 |
| rplV | MGM1_0760 | P47402 | Q8EUB8 | P75575 | Q9PQQ5 | B5ZB45 | F0V3N5 | O52337 | E8ZJZ4 | G4ELH5 |
| rpmA | MGM1_1520 | P47476 | Q8EVW5 | P75458 | Q9PQT2 | B5ZB18 | F0V3B3 | Q7NBB0 | E8ZKT1 | G4EM87 |
| rplB | MGM1_0740 | P47400 | Q8EUB6 | P75577 | Q9PQQ7 | B5ZB43 | F0V3N3 | O52335 | E8ZJZ6 | G4ELH7 |
| rplC | MGM1_0710 | P47397 | Q8EUB3 | P75580 | Q9PQR0 | B5ZB40 | F0V3M9 | O52332 | E8ZJZ9 | G4ELI0 |
| rplD2 | MGM1_0720 | P47398 | Q8EUB4 | P75579 | Q9PQQ9 | B5ZB41 | F0V3N0 | O52333 | E8ZJZ8 | G4ELH9 |
| rplE | MGM1_0830 | P47409 | Q8EUC5 | Q50306 | Q9PQP8 | B5ZB52 | F0V3P2 | O52344 | E8ZJY7 | G4ELG8 |
| rplF | MGM1_0860 | P47412 | Q8EUC8 | Q50303 | Q9PQP5 | B5ZB55 | F0V3P5 | O52347 | E8ZJY4 | G4ELG5 |
| ykqC | MGM1_5960 | P47385 | Q8EVN2 | P75497 | Q9PPR9 | B5ZC81 | F0V1I3 | Q7NC60 | E8ZK97 | G4EM16 |
| rpoA | MGM1_0980 | P47423 | Q8EUD7 | Q50295 | Q9PQN4 | B5ZB66 | F0V1F1 | Q9RDV6 | E8ZJX3 | G4ELF4 |
| rpsJ | MGM1_0700 | P47396 | Q8EUB2 | P75581 | Q9PQR1 | B5ZB39 | F0V3M8 | O52331 | E8ZK00 | G4ELI1 |
| rpsL | MGM1_0190 | P47333 | Q8EX21 | P75546 | Q9PPW5 | B5ZC34 | F0V1H1 | Q7NAV1 | E8ZGH8 | G4ENC4 |
| rpsM | MGM1_0950 | P47421 | Q8EUD6 | Q50297 | Q9PQN6 | B5ZB64 | F0V1E9 | Q9RDV8 | E8ZJX5 | G4ELF6 |
| rpsR | MGM1_3010 | P47338 | Q8EWT5 | P75541 | Q9PPT8 | B5ZC59 | F0V2P2 | Q7NAM3 | E8ZGJ5 | G4ELW4 |
| rpsS | MGM1_0750 | P47401 | Q8EUB7 | P75576 | Q9PQQ6 | B5ZB44 | F0V3N4 | O52336 | E8ZJZ5 | G4ELH6 |
| rpsB | MGM1_5210 | P47316 | Q8EUG1 | P75560 | Q9PRB9 | B5ZAJ9 | F0V3H2 | Q7NAW2 | E8ZJW9 | G4ELJ1 |
| rpsC | MGM1_0770 | P47403 | Q8EUC1 | P41205 | Q9PQQ4 | B5ZB46 | F0V3N6 | O52338 | E8ZJZ3 | G4ELH4 |
| rpsD | MGM1_0150 | P47553 | Q8EV12 | P46775 | Q9PPZ2 | B5ZC02 | F0V3L6 | Q7NAV0 | E8ZKF1 | G4EMA3 |
| rpsE | MGM1_0880 | P47414 | Q8EUD0 | Q50301 | Q9PQP3 | B5ZB57 | F0V3P7 | O52349 | E8ZJY2 | G4ELG3 |
| rpsG | MGM1_0180 | P47334 | Q8EX20 | P75545 | Q9PPW6 | B5ZC33 | F0V1H2 | Q7NAV2 | E8ZK83 | G4ENC3 |
| rpsH | MGM1_0850 | P47411 | Q8EUC7 | Q50304 | Q9PQP6 | B5ZB54 | F0V3P4 | O52346 | E8ZJY5 | G4ELG6 |
| rpsI | MGM1_5450 | P47656 | Q8EWW8 | P75179 | Q9PPR3 | B5ZC90 | F0V3M4 | Q7NBH4 | E8ZIX3 | G4ELN8 |
| rsmA | MGM1_6080 | P47701 | Q8EU92 | P75113 | Q9PPN8 | B5ZCB6 | F0V2M0 | Q7NC69 | E8ZK79 | G4ELA7 |
| secA | MGM1_2690 | P47318 | Q8EVL6 | P75559 | Q9PR25 | B5ZAV1 | F0V295 | Q7NC50 | E8ZKA1 | G4ENP5 |
| secY | MGM1_0900 | P47416 | Q8EUD2 | Q59548 | Q9PQP1 | B5ZB59 | F0V3P9 | O52351 | E8ZJY0 | G4ELG1 |
| alaS | MGM1_4050 | P47534 | Q8EUR6 | P75368 | Q9PQC2 | B5ZBI1 | F0V3C4 | Q7NBY8 | E8ZKC6 | G4EM02 |
| pheS | MGM1_0480 | P47436 | Q8EUJ8 | P75564 | Q9PQ32 | B5ZBV3 | F0V2U0 | Q7NBB8 | E8ZK48 | G4ELU0 |
| hisS | MGM1_2230 | P47281 | Q8EWB8 | P75069 | Q9PQK6 | B5ZB96 | F0V3I1 | Q7NBS9 | E8ZJV9 | G4ELN3 |
| ileS | MGM1_4410 | P47587 | Q8EUN9 | P75258 | Q9PQ79 | B5ZBQ7 | F0V3I7 | Q7NBG0 | E8ZKR3 | G4ELT6 |
| leuS | MGM1_1780 | P47508 | Q8EW18 | P75398 | Q9PQC0 | B5ZBI3 | F0V3I3 | Q7NB47 | E8ZJV5 | G4EMJ6 |
| metS | MGM1_5640 | P47267 | Q8EUI8 | P75091 | Q9PQU6 | B5ZB02 | F0V2I0 | Q7NBV3 | E8ZGG8 | G4EMU5 |
| asnS | MGM1_2000 | P47359 | Q8EW03 | P75521 | Q9PQC6 | B5ZBH7 | F0V3C8 | Q7NBM9 | E8ZKC3 | G4EMZ4 |
| serS | MGM1_4830 | P47251 | Q8EW01 | P75107 | Q9PR38 | B5ZAT6 | F0V2L7 | Q7NAD6 | E8ZK75 | G4EMZ2 |
| oppF-valS | MGM1_2740 | P47576 | Q8EX08 | P75304 | Q9PQM4 | B5ZB77 | F0V1M0 | Q7NAR7 | E8ZKF8 | G4ELB8 |
| trpS | MGM1_3900 | P47372 | Q8EVV1 | P75510 | Q9PQW8 | B5ZAY1 | F0V2Q2 | Q7NAT8 | E8ZK67 | G4ENR3 |
| tyrS | MGM1_3170 | P47693 | Q8EUX2 | P75122 | Q9PR27 | B5ZAU9 | F0V2N8 | Q7NBH9 | E8ZGJ1 | G4EM42 |
| ychF | MGM1_0040 | P47270 | Q8EU98 | P75088 | Q9PPP4 | B5ZCB0 | F0V2S2 | F8WJY6 | E8ZK66 | G4ELA1 |
| hit | MGM1_0250 | P47378 | Q8EW55 | P75504 | Q9PQL9 | B5ZB82 | F0V1G9 | Q7NAX8 | E8ZIY9 | G4EML1 |
