# Supplementary material for: An Emerging Mycoplasma Associated with Trichomoniasis, Vaginal Infection and Disease
Source: PLoS One. 2014 Oct 22;9(10):e110943. doi: 10.1371/journal.pone.0110943 (PMC4206474; doi:10.1371/journal.pone.0110943)
Supplement: Table S6 — Spiroplasma, Phytoplasma and Outgroup orthologs. (DOCX) [file pone.0110943.s010.docx]

**Table S6. Spiroplasma, Phytoplasma and Outgroup orthologs.**

| **Gene** | ***Onion yellows phytoplasma* OY-M** | ***Mesoplasma florum* L1** | ***Mycoplasma mycoides* subsp. Mycoides SC str. PG1** | ***Aster yellows witches’-broom phytoplasma* AYWB** | ***Lactobacillus gasseri* ATCC 33323** | ***Mycoplasma capricolum* subsp. Capricolum ATCC 27343** | ***Acholeplasma laidlawii* PG-8A** | ***Phytoplasma mali* AT** | ***Phytoplasma australiense*** | ***Mycoplasma leachii* PG50** |
| --- | --- | --- | --- | --- | --- | --- | --- | --- | --- | --- |
| engA | Q6YQY3 | Q6F1R7 | Q6MTJ6 | Q2NIZ5 | Q043U0 | Q2SRR7 | A9NGK7 | B3R0E4 | B1VAZ1 | E4PUI5 |
| dnaJ | Q6YPM2 | Q6F150 | Q6MT07 | Q2NK64 | Q044A8 | P71500 | A9NFN9 | B3QZK7 | B1VAB5 | E4PU03 |
| cbiO2 | Q6YR39 | Q6F1W5 | Q6MSQ1 | Q2NIT5 | Q045Z8 | Q2SRI1 | A9NEG9 | B3R019 | B1V978 | E4PUT4 |
| tuf | Q6YQV8 | Q6F0J5 | Q6MU81 | Q2NJ20 | Q042T5 | Q2SSW8 | A9NEN4 | B3QZH5 | B1VAM1 | E4PTH5 |
| gyrA | Q6YQ76 | Q6F2B2 | Q6MUM1 | Q2NJM2 | Q047E9 | Q2ST76 | A9NE70 | B3R0L9 | B1V9B7 | E4PT53 |
| hrcA | Q6YPL9 | Q6F147 | Q6MT04 | Q2NK67 | Q044B1 | P71498 | A9NFN6 | B3QZK4 | B1VAB8 | E4PU00 |
| infB | Q6YR66 | Q6F1H1 | Q6MTQ0 | Q2NIQ6 | Q044B7 | Q2SSE6 | A9NF12 | B3QZW5 | B1VAG9 | E4PTW6 |
| adk | Q6YR01 | Q6F1X3 | Q6MSP5 | Q2NIX5 | Q046A5 | P10251 | A9NEF4 | B3R054 | B1VAC7 | E4PUU2 |
| gmk | P60554 | Q6F1S1 | Q6MU25 | Q2NIH1 | Q044H3 | Q2SSR8 | A9NEZ6 | B3R0Q0 | B1V908 | E4PTM1 |
| mnmA | Q6YR90 | Q6F152 | Q6MTG1 | Q2NIM9 | Q042R4 | Q2SRW8 | A9NFP7 | B3R0K5 | B1VAX7 | E4PUE6 |
| nusA | Q6YR68 | Q6F1H4 | Q6MTP7 | Q2NIQ4 | Q044C0 | Q2SSE3 | A9NF09 | B3QZW3 | B1VAG7 | E4PTW9 |
| pth | Q6YR64 | Q6F240 | Q6MS28 | Q2NIQ9 | Q046E4 | Q2ST16 | A9NE82 | B3QZM3 | B1VAH1 | E4PTC0 |
| rplK | P62439 | Q6F0K7 | P62437 | Q2NJ11 | Q045W1 | Q2ST53 | A9NEL2 | B3QZG6 | B1VAN0 | E4PT78 |
| rplM | Q6YR78 | Q6F0X2 | Q6MSQ6 | Q2NIP4 | Q045Z4 | Q2SRI6 | A9NEI3 | B3QZY2 | B1VA57 | E4PUS9 |
| rplN | Q6YR11 | Q6F1Y4 | Q6MSN5 | Q2NIW4 | Q046B5 | P10137 | A9NEE3 | B3R001 | B1VAD8 | E4PUV2 |
| rplO | Q6YR03 | Q6F1X6 | Q6MSP3 | Q2NIX3 | Q046A7 | P10138 | A9NEF2 | B3R010 | B1VAC9 | E4PUU4 |
| rplP | Q6YR14 | Q6F1Y7 | Q6MSN2 | Q2NIW1 | Q046B8 | P02415 | A9NEE0 | B3QZZ8 | B1VAE1 | E4PUV5 |
| rplQ | Q6YQZ4 | Q6F1W6 | Q6MSQ0 | Q2NIY2 | Q045Z9 | Q48980 | A9NEG1 | B3R018 | B1VAC0 | E4PUT5 |
| rplA | Q6YQW6 | Q6F0K8 | Q6MRY7 | Q2NJ12 | Q045W0 | Q2ST52 | A9NEL3 | B3QZG7 | B1VAM9 | E4PT79 |
| rplT | Q6YPI5 | Q6F1S6 | Q6MU20 | Q2NIG6 | Q041V3 | Q2SSS3 | A9NGH5 | B3R0P6 | B1V900 | E4PTL6 |
| rplV | Q6YR16 | Q6F1Y9 | Q6MSN0 | Q2NIV9 | Q046C0 | P10139 | A9NED8 | B3QZZ6 | Q6W943 | E4PUV7 |
| rpmA | Q6YRC7 | Q6F125 | Q6MT50 | Q2NIJ9 | Q044I5 | Q2SS74 | A9NF56 | B3QZT2 | B1VAF3 | E4PU37 |
| rplB | P60402 | Q6F1Z1 | Q6MSM8 | Q2NIV7 | Q046C2 | P10133 | A9NED6 | B3QZZ4 | B1VAE5 | E4PUV9 |
| rplC | P60455 | Q6F1Z4 | Q6MSM6 | Q2NIV4 | Q046C5 | P10134 | A9NED3 | B3QZZ1 | B1VAE8 | E4PUW2 |
| rplD2 | P61067 | Q6F1Z3 | P61065 | Q2NIV5 | Q046C4 | P10135 | A9NED4 | B3QZZ2 | B1VAE7 | E4PUW1 |
| rplE | Q6YR10 | Q6F1Y2 | Q6MSN8 | Q2NIW6 | Q046B3 | P10136 | A9NEE5 | B3R003 | B1VAD6 | E4PUV0 |
| rplF | Q6YR07 | Q6F1X9 | Q6MSP0 | Q2NIW9 | Q046B1 | P04448 | A9NEE8 | B3R006 | B1VAD3 | E4PUU7 |
| ykqC | Q6YPU2 | Q6F0P5 | Q6MSU8 | Q2NK25 | Q042R9 | Q2SRM2 | A9NF01 | B3QZL1 | B1V8V9 | E4PUN6 |
| rpoA | Q6YQZ5 | Q6F1W7 | Q6MSP9 | Q2NIY1 | Q046A0 | Q2SRH9 | A9NEG0 | B3R017 | B1VAC1 | E4PUT6 |
| rpsJ | Q6YR19 | Q6F1Z5 | Q6MSM5 | Q2NIV2 | Q046C6 | P10129 | A9NED2 | B3QZZ0 | B1VAE9 | E4PUW3 |
| rpsL | Q6YQW1 | Q6F0J2 | Q8VMU2 | Q2NJ17 | Q046C9 | Q2SSX1 | A9NEN1 | B3QZH2 | B1VAM4 | E4PTH2 |
| rpsM | Q6YQZ7 | Q6F1W9 | Q6MSP7 | Q2NIX9 | Q046A2 | Q2SRH7 | A9NEF8 | B3R015 | B1VAC3 | E4PUT8 |
| rpsR | Q6YRK3 | Q6F235 | Q6MUK6 | Q2NKC1 | Q047E6 | Q2ST89 | A9NEP1 | B3R0K1 | B1V953 | E4PT06 |
| rpsS | Q6YR17 | Q6F1Z0 | Q6MSM9 | Q2NIV8 | Q046C1 | P10132 | A9NED7 | B3QZZ5 | B1VAE4 | E4PUV8 |
| rpsB | Q6YR51 | Q6F0Q4 | Q6MT08 | Q2NIS2 | Q044D0 | Q2SSA8 | A9NHC6 | B3QZN2 | B1VA80 | E4PU04 |
| rpsC | Q6YR15 | P0DJ10 | Q6MSN1 | Q2NIW0 | Q046B9 | P02353 | A9NED9 | B3QZZ7 | B1VAE2 | E4PUV6 |
| rpsD | Q6YPY9 | Q6F0M9 | Q6MTW7 | Q2NJN5 | Q042M7 | Q2SSN9 | A9NHG9 | B3QZW0 | B1V8Z1 | E4PTQ7 |
| rpsE | Q6YR05 | Q6F1X7 | Q6MSP2 | Q2NIX1 | Q046A9 | P10128 | A9NEF0 | B3R008 | B1VAD1 | E4PUU5 |
| rpsG | Q6YQW0 | Q6F0J3 | Q8VMU1 | Q2NJ18 | Q046C8 | Q2SSX0 | A9NEN2 | B3QZH3 | B1VAM3 | E4PTH3 |
| rpsH | Q6YR08 | Q6F1Y0 | Q6MSN9 | Q2NIW8 | Q046B2 | P04446 | A9NEE7 | B3R005 | B1VAD4 | E4PUU8 |
| rpsI | Q6YR77 | Q6F0X3 | Q6MSQ7 | Q2NIP5 | Q045Z3 | Q2SRI7 | A9NEI4 | B3QZY3 | B1VA58 | E4PUS8 |
| rsmA | Q6YPJ4 | Q6F2B4 | Q6MUM4 | Q2NIH8 | Q046K5 | P43038 | A9NE79 | B3QZM6 | B1V9I5 | E4PSX9 |
| secA | Q6YQA1 | Q6F260 | Q6MUE3 | Q2NJH2 | Q042C9 | Q2ST71 | A9NF22 | B3R0C3 | B1VAB2 | E4PT57 |
| secY | Q6YR02 | Q6F1X5 | Q6MSP4 | Q2NIX4 | Q046A6 | P10250 | A9NEF3 | B3R011 | B1VAC8 | E4PUU3 |
| alaS | Q6YQR8 | Q6F0K3 | P61704 | Q2NJ60 | Q045P6 | Q2SSW4 | A9NGN9 | B3QZP8 | B1VA70 | E4PTI1 |
| pheS | Q6YPX9 | Q6F165 | Q6MT17 | Q2NJZ3 | Q041W8 | Q2SSA0 | A9NEU5 | B3R0A3 | B1VA93 | E4PU12 |
| hisS | P60918 | Q6F199 | P62372 | Q2NIN2 | Q043X4 | Q2SSF4 | A9NGF8 | B3QZS0 | B1VA46 | E4PTV8 |
| ileS | Q6YR34 | Q6F175 | Q6MT28 | Q2NIU1 | Q042Q7 | Q2SS90 | A9NG38 | B3QZQ2 | B1V9Q9 | E4PU22 |
| leuS | Q6YPI9 | Q6F0X5 | Q6MSR0 | Q2NIH0 | Q045L5 | Q2SRI9 | A9NET8 | B3R0P9 | B1V907 | E4PUS6 |
| metS | Q6YQZ0 | Q6F281 | Q6MUL6 | Q2NIY7 | Q046K8 | Q2ST80 | A9NEH8 | B3QZF6 | B1V931 | E4PT48 |
| asnS | Q6YPH3 | Q6F0Y5 | Q6MUF2 | Q2NIF5 | Q043G9 | Q2SR42 | A9NE58 | B3R0I1 | B1V8X4 | E4PSR9 |
| serS | Q6YRD2 | Q6F2A0 | Q6MUG5 | Q2NIJ4 | Q041F5 | Q2SR32 | A9NE72 | B3R0F5 | B1VAF7 | E4PST0 |
| oppF-valS | Q6YRJ6 | Q6F1B1 | Q6MTU2 | Q2NKB4 | Q042N4 | Q2SSL9 | A9NFM9 | B3QZY8 | B1V945 | E4PTT0 |
| trpS | Q6YQA9 | Q6F1S4 | Q6MTP1 | Q2NJG4 | Q046L1 | Q2SSD7 | A9NHF1 | B3R0B5 | B1VAA3 | E4PTX5 |
| tyrS | Q6YQA6 | Q6F0M8 | Q6MST3 | Q2NJG7 | Q046N3 | Q2SRK6 | A9NHF4 | B3R0D7 | B1VAA7 | E4PUQ7 |
| ychF | Q6YR35 | Q6F0F7 | Q6MS65 | Q2NIU0 | Q040U5 | Q2SR60 | A9NG41 | B3QZQ1 | B1V9R0 | E4PSQ0 |
| hit | Q6YPU5 | Q6F1M1 | Q6MTB0 | Q2NK22 | Q041R9 | Q2SS17 | A9NE75 | B3R0F4 | B1VAR6 | E4PU94 |
